# Unraveling the Intestinal Microbiota Conundrum in Allogeneic Hematopoietic Stem Cell Transplantation: Fingerprints, Clinical Implications and Future Directions

**DOI:** 10.3390/jcm14196874

**Published:** 2025-09-28

**Authors:** Alexandre Soares Ferreira Júnior, Bianca Fernanda Rodrigues da Silva, Jefferson Luiz da Silva, Mariana Trovão da Silva, João Victor Piccolo Feliciano, Iago Colturato, George Maurício Navarro Barros, Phillip Scheinberg, Nelson Jen An Chao, Gislane Lelis Vilela de Oliveira

**Affiliations:** 1 Department of Genetics, Microbiology and Immunology, Institute of Biosciences, Sao Paulo State University, Botucatu 18618-970, SP, Brazil; alexandre.soares@unesp.br (A.S.F.J.);; 2 Fundação Faculdade Regional de Medicina de São José do Rio Preto, São José do Rio Preto 15090-000, SP, Brazil; joao.feliciano@hospitaldebase.com.br; 3 Hospital Amaral Carvalho, Jaú 17210-070, SP, Brazil; 4 Fundação Pio XII, Hospital de Câncer de Barretos, Barretos 14784-400, SP, Brazil; 5 Division of Hematology, Hospital A Beneficência Portuguesa, São Paulo 01323-001, SP, Brazil; 6 Department of Medicine, Division of Hematologic Malignancies and Cellular Therapy, Duke University, Durham, NC 27710, USA

**Keywords:** allogeneic hematopoietic stem cell transplantation, Graft-versus-host disease, intestinal microbiota diversity, intestinal dominance, clinical outcomes

## Abstract

Intestinal dysbiosis represents a critical determinant of clinical outcomes in patients undergoing allogeneic hematopoietic stem cell transplantation (allo-HSCT). Distinct microbiota patterns represent potential prognostic biomarkers and therapeutic targets. However, the exponential growth in microbiota research and analytical complexity has created significant interpretive challenges for clinicians. This review provides a synthesis of current literature examining microbiota fingerprints and their clinical implications. We analyzed key studies evaluating the clinical implications of intestinal microbiota fingerprints in allo-HSCT. Additionally, we examined current therapeutic strategies for microbiota modulation and approaches for translating research findings into clinical practice. We identified three major microbiota fingerprints: (1) decreased intestinal microbiota diversity, (2) reduced abundance of short-chain fatty acid-producing bacteria, and (3) *Enterococcus* domination. These fingerprints are associated with critical clinical outcomes including overall survival, Graft-versus-host disease, transplant-related mortality, and infection-related complications. While fecal microbiota transplantation and dietary interventions appear promising, current studies suffer from limited sample sizes and lack standardized protocols. Despite significant advances in microbiota research, biological, methodological, and logistical challenges continue to hinder its clinical translation. Understanding microbiota fingerprints represents a promising avenue for improving allo-HSCT outcomes. However, successful clinical implementation requires standardized methodologies, mechanistic studies, and multi-center collaborations to translate research into actionable clinical tools.

## 1. Introduction

Allogeneic hematopoietic stem cell transplantation (allo-HSCT) is a potentially curative therapy for several malignant and non-malignant diseases. However, its effectiveness remains limited due to life-threatening complications, including neutropenic fever, relapse and acute graft versus host disease (aGvHD) [1,2,3,4,5,6]. Acute GvHD remains the leading cause of non-relapse mortality, affecting up to 70% of patients [1,2,3,4]. Relapse remains the primary cause of treatment failure, with incidence reported in up to 40% of cases [6,7]. Similarly, neutropenic fever is highly prevalent, occurring in up to 80% of patients—particularly during the pre-engraftment period [5]. Thus, allo-HSCT is still associated with considerable morbidity. During the allo-HSCT, the intestinal microbiota has emerged as a key player that can shape the development of these poor outcomes. Specific patterns of intestinal microbiota disruption—often referred to as “microbiota fingerprints”—have been linked to these poor outcomes. Consequently, understanding these microbiota fingerprints is increasingly recognized as crucial for predicting outcomes and developing new therapeutic interventions in the transplantation context.

In recent years, there has been a significant increase in studies investigating the role of the intestinal microbiota in allo-HSCT. These studies consistently demonstrate the strong link between intestinal microbiota disruptions and poor clinical outcomes. Nevertheless, the growing volume of data and the complexity of microbiota analysis may pose significant barriers to understanding how these fingerprints impact allo-HSCT patients. Additionally, prior allo-HSCT reviews have focused narrowly on analyzing the relationships of intestinal microbiota with specific diseases and/or complications, failing to provide a comprehensive analysis of clinical implications. Furthermore, most microbiota studies are observational, mechanistically inconsistent and not easily translated into clinical practice. In this review, we aim to clarify this conundrum by summarizing key studies that have evaluated intestinal microbiota fingerprints and their clinical implications for patients undergoing allo-HSCT. After unraveling this conundrum, we will also provide a critical overview of challenges and strategies to: **(1)** modulate the intestinal microbiota, and **(2)** facilitate the integration of intestinal microbiota research into clinical practice. The scientific novelty of this review lies in systematizing current data on microbiota fingerprints and synthesizing them into a comprehensive, clinically meaningful framework that bridges microbiota research with clinical practice.

To conduct this narrative review, an electronic search was performed using PubMed. The search aimed to identify key studies analyzing the relationship between intestinal microbiota and allo-HSCT outcomes. The following keywords were used: “hematopoietic stem cell transplantation”, “gut microbiome”, “microbiota”, “Graft-versus-host disease”, “fecal microbiota transplantation”, “probiotics”, “prebiotics”, and “synbiotics”. Only key articles providing detailed data regarding intestinal microbiota fingerprints, strategies to modulate intestinal microbiota and allo-HSCT outcomes were included. The search strategy was supplemented by manually reviewing the reference lists of included articles to identify additional relevant studies.

### Brief Overview of Human Intestinal Microbiota

The intestinal microbiota comprises a set of commensal microorganisms essential for maintaining human health, including bacteria, fungi, and viruses, which colonize the entire gastrointestinal tract and coexist with the host [8,9,10]. In healthy adults, the intestinal microbiota is predominantly composed of members from the Bacillota and Bacteroidota phyla, while other microorganisms are present in lower abundance. The intestinal microbiota plays a pivotal role in human physiology, contributing to **(1)** host homeostasis, **(2)** nutrient metabolism, **(3)** vitamin synthesis, **(4)** immune system modulation, and **(5)** production of metabolites.

The intestinal microbiota has been recognized as a key factor in driving disease development. Intestinal dysbiosis has been linked to inflammatory bowel disease, irritable bowel syndrome, autoimmune diseases and metabolic disorders [8,9,10]. Intestinal dysbiosis has also been linked to cancer and responses to both immunotherapies and chemotherapies [8,11]. Given the exponential growth of microbiota research and its expanding associations with disease development, severity and treatment responses, it is increasingly important for clinicians to integrate microbiota science into both research and clinical practice. In the following sections, we will highlight the most important patterns of intestinal dysbiosis in the allo-HSCT context and their impacts on clinical outcomes.

## 2. The Dynamics of Intestinal Microbiota Through the Patient Journey

**Figure 1:**
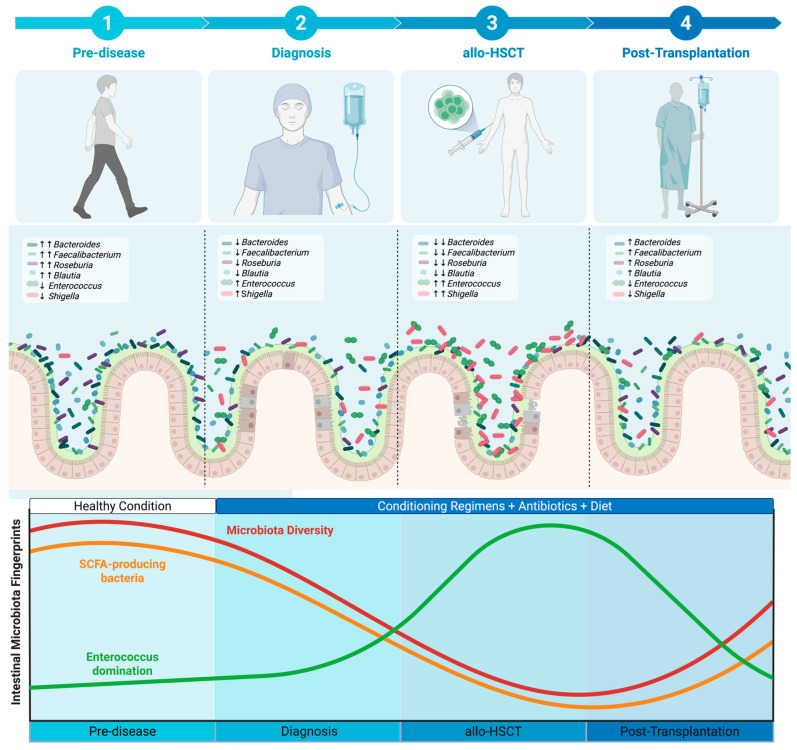
The dynamics of the three intestinal microbiota fingerprints (microbiota diversity, SCFA-producing bacteria and *Enterococcus* intestinal domination) through the allo-HSCT journey. Allo-HSCT = allogeneic hematopoietic stem cell transplantation; SCFA = Short-chain fatty acids; ↑ = increase; ↓ = decrease. Created with BioRender.com, Soares Ferreira Junior A. (2025) https://BioRender.com/8du0b87 (accessed on 19 September 2025).

**Figure 2:**
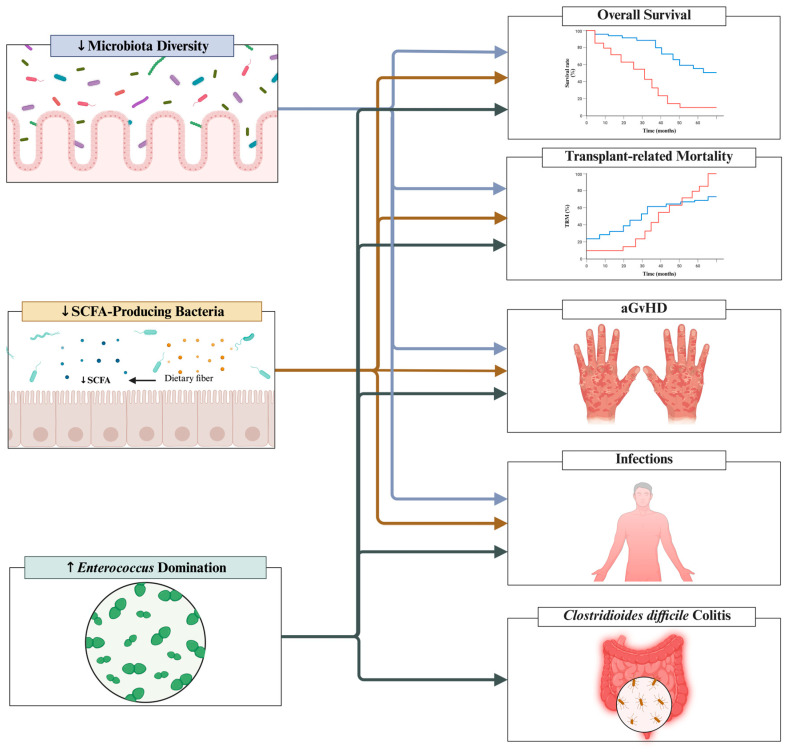
Associations among intestinal microbiota fingerprints (microbiota diversity, SCFA-producing bacteria and *Enterococcus* intestinal domination) and clinical outcomes (overall survival, transplant-related mortality, aGvHD, infections and *Clostridioides difficile* colitis). Red and blue lines in the overall survival and transplant-related mortality figure correspond to patients with high and low intestinal diversity, respectively. aGvHD = acute graft versus host disease; SCFAs = Short-chain fatty acids; ↑ = increase; ↓ = decrease. Created with BioRender.com, Soares Ferreira Junior A. (2025) https://BioRender.com/1f3oh4x (accessed on 19 September 2025).

The patient journey until the allo-HSCT is marked by dynamic and progressive disruptions in the intestinal microbiota. The intestinal microbiota may start to change to a disease-associated layout after the diagnosis of the underlying condition [12,13]. Several studies have demonstrated key features of intestinal dysbiosis even prior to the allo-HSCT [14,15,16,17,18,19]. The extent of intestinal microbiota disruption during allo-HSCT can be so severe that the re-establishment of homeostasis or eubiosis condition may be markedly delayed, or in some cases, may not be achieved at all [15,17]. Overall, through the patient journey until completing the allo-HSCT procedure, key intestinal microbiota fingerprints may emerge. The most important intestinal microbiota fingerprints identified across studies are: **(1)** decreased intestinal microbiota diversity [16,17,20,21,22,23,24,25,26,27]; **(2)** decreased abundance of SCFA (short-chain fatty acids)-producing bacteria [14,25,28,29,30]; and **(3)** *Enterococcus* intestinal domination (see Figure 1) [22,24,31,32,33,34]. Besides these consistent fingerprints, it is important to note that studies show variable prognostic value for specific bacterial taxa [20,21,35,36]. Multiple studies have identified several taxa associated with either protection or increased risk of poor outcomes. This substantial heterogeneity may result from the lack of methodological standardization across microbiota studies, including differences in sample collection techniques, collection timing, and DNA sequencing methodologies. Due to the lack of consensus and standardization in this area, specific taxa will not be the focus of this review. Instead, we concentrate on the three microbiota fingerprints with the most robust and reproducible evidence linking them to relevant clinical outcomes. These outcomes include overall survival, transplantation-related mortality, aGvHD, infections, and *Clostridioides difficile* colitis (see Figure 2). Given that these intestinal dysbiosis fingerprints have prognostic significance, understanding the factors driving them is critical for improving patient outcomes.

Key factors driving these intestinal dysbiosis fingerprints through the patient journey include chemotherapies, dietary changes and the use of broad-spectrum antibiotics [14,15,20,21,30,36,37,38,39,40]. The relationship between antibiotic exposure and intestinal dysbiosis was demonstrated in a study including 96 patients [20]. When compared to patients (*n* = 34) without antibiotic exposure, patients (*n* = 62) receiving any antibiotic in the 3 months prior to allo-HSCT had significantly lower alpha diversity (41.5 ± 26.54 vs. 61.26 ± 25.93; *p* = 0.001) [20]. Similar findings were also reported in a study including 57 patients undergoing allo-HSCT—prior antibiotic use was significantly associated with lower bacterial diversity (*p* = 0.003) [16]. In this study, other factors associated with intestinal dysbiosis were **(1)** severe underlying hematologic disease (*p* < 0.0001); **(2)** CMV (cytomegalovirus) seropositivity (*p* = 0.006), **(3)** gastrointestinal or hepatic comorbidities (*p* = 0.004), and **(4)** recent microbial infection (*p* = 0.006) [16]. The impact of different conditioning regimens on the intestinal microbiota has also been evaluated. For example, in a study involving 96 patients undergoing allo-HSCT, those who received myeloablative conditioning exhibited distinct shifts in microbiota composition compared to patients who received reduced-intensity regimens [20]. These findings suggest that several factors contribute to the development of intestinal dysbiosis during allo-HSCT. Nevertheless, whether these factors act individually or synergistically to drive these intestinal dysbiosis fingerprints requires future studies. With a better understanding of the potential drivers of these fingerprints, the following sections will explore the key moments at which these patterns emerge and their potential implications for clinical outcomes.

## 3. Intestinal Microbiota Fingerprints Prior to Allo-HSCT

In patients undergoing allo-HSCT, there is extensive evidence suggesting that the intestinal microbiota is disrupted even prior to the transplantation [14,15,16,17,18,19,41]. Prior to allo-HSCT, the most important intestinal dysbiosis fingerprint is decreased intestinal diversity [14,15,19,20,37,38,39,40,41]. Studies have also shown that patients already exhibit a distinct microbiota composition prior to undergoing allo-HSCT (see Supplementary Table S1) [14,15,19,20,37,38,39,40]. When compared to a control group (paired HLA [Human Leukocyte Antigen]-matched sibling donors), patients (*n* = 57) had lower bacterial diversity (*p* = 0.0002) and different phylogenetic membership (*p* = 0.001) with increased relative abundances of facultative anaerobic bacteria (such as *Enterococcaceae* and *Streptococcaceae*) [16]. Furthermore, patients had significantly lower abundance of butyrate-producing bacteria (which produce a key metabolite that helps maintain a healthy gut) compared to healthy volunteers. These bacteria included *Anaerostipes* (*p* = 0.036), *Butyricimonas* (*p* = 0.041), *Coprococcus* (*p* < 0.001), *Faecalibacterium* (*p* = 0.014), and *Lachnospiraceae* (*p* < 0.001) [14]. Similarly, fecal samples from 606 patients showed that the intestinal microbiota was significantly different from healthy adult volunteers and subjects from the Human Microbiome Project [17]. In this study, patients undergoing allo-HSCT had lower intestinal diversity (*p* < 0.001) and a distinct microbiota composition based on enterotype (*p* < 0.001) [17]. Taken together, these and other studies in the literature suggest that intestinal dysbiosis exists even prior to allo-HSCT [14,15,16,17,18,19]. Given that these intestinal dysbiosis fingerprints have prognostic significance, the next step is to clarify their implication for patient outcomes.

These early intestinal dysbiosis fingerprints are not only present but can significantly shape the course and prognosis of patients undergoing allo-HSCT. These early fingerprints can contribute to the following outcomes: **(1)** overall survival [16,17,20,21]; **(2)** transplantation-related mortality [17]; **(3)** aGvHD [20,21,35,36]; **(4)** and infections [20]. Among the fingerprints, the most evaluated prior to allo-HSCT is the intestinal microbiota diversity—an index that measures the variety (richness) and balance (evenness) of bacteria living in the gastrointestinal tract (see Table 1). Across studies, a consistent finding is that decreased intestinal microbiota diversity prior to allo-HSCT is associated with poor outcomes, such as increased mortality and GvHD [16,17,21,35]. Among the available studies, the most robust in terms of statistical power and sample size was an international multi-center study involving 606 patients. In this study, higher intestinal diversity prior to the transplant was associated with significant reductions in mortality. Specifically, it reduced the risk of death by 59% (HR [Hazard Ratio] 0.41; 95% CI [Confidence Interval] 0.24–0.71) and transplant-related mortality by 56% (HR 0.44; 95% CI 0.22–0.87) [17].

The other fingerprint relates to specific bacterial compositions prior to allo-HSCT that are associated with either protection against or increased risk for poor outcomes (see Supplementary Table S2). For example, higher abundance of *Blautia*, which produces SCFA and promotes gut homeostasis, was associated with lower risk of aGvHD development in two studies [21,35]. Given the prognostic significance of these intestinal dysbiosis fingerprints, future studies should evaluate strategies to incorporate them into risk stratification tools that can be used in clinical practice.

## 4. Intestinal Microbiota Fingerprints During Allo-HSCT

The available literature has demonstrated that during allo-HSCT, the already compromised intestinal microbiota undergoes further dysbiosis (see Figure 1) [20,21,22,23,24,25,26]. As the intestinal microbiota changes, all three key fingerprints emerge: **(1)** decreased intestinal diversity [20,21,22,23,24,25,26,27,42]; **(2)** decreased abundance of SCFA-producing bacteria [25,28]; and **(3)** intestinal domination by a single taxon [22,24,31,32,33,34]. In the following subsections, we will review pivotal studies that have both described these fingerprints and examined their clinical implications.

### 4.1. Intestinal Diversity and Implications to Clinical Outcomes

During allo-HSCT, the intestinal diversity continues to decline and may not return to baseline levels [17,20,21,22,23,24,25,27,43]. In a previous study including 96 patients, stool samples were collected at three timepoints: **(1)** baseline (prior to the conditioning regimen), **(2)** D (day) + 10, and **(3)** D + 30 [20]. Compared to baseline, samples collected at both D + 10 and D + 30 showed a significant reduction in intestinal diversity (D + 10: 4.65 ± 1.36 vs. 3.08 ± 1.77; *p* < 0.001; D + 30: 4.65 ± 1.36 vs. 2.62 ± 1.62; *p* < 0.001) [20]. This study also identified a significant reduction in intestinal diversity in samples collected at D + 30 when compared to D + 10 samples (2.62 ± 1.62 vs. 3.08 ± 1.77; *p* = 0.020) [20]. Similar findings were also reported in a study involving 100 patients. Stool samples were collected at four timepoints: **(1)** baseline (prior to the conditioning regimen), **(2)** around the day of stem cell infusion (D − 4 to D0), **(3)** engraftment (D + 4 to D + 28), and **(4)** late post-HSCT (after D + 28) [24]. This study showed a significant reduction in intestinal diversity when comparing baseline samples to those collected around the day of stem cell infusion (*p* < 0.05) and engraftment (*p* < 0.01) [24]. Although intestinal diversity significantly increased in late post-HSCT samples compared to those collected during engraftment (*p* < 0.05), it remained below baseline levels [24]. Taken together, these and other studies demonstrate that intestinal diversity reaches its lowest values within 30 days after the allo-HSCT and gradually increases thereafter [17,20,21,22,23,24,25,27,43]. However, it often remains below baseline even in samples collected 100 days post-transplant (see Supplementary Table S3). Thus, future studies are needed to explore strategies to preserve and restore intestinal diversity over the allo-HSCT course.

Strategies to preserve and restore the intestinal diversity have become a priority as research has shown it to be a key prognostic factor in patients undergoing allo-HSCT [15,44,45]. In the literature, intestinal diversity during allo-HSCT has been linked to overall survival [17,23,24], aGvHD [20,26,28,41,46], and transplantation-related mortality (see Table 2) [23,24,41]. In most of the studies, lower intestinal diversity in samples collected at the engraftment period has been associated with these poor outcomes [17,20,23,24,28,41,46]. Furthermore, lower intestinal diversity at the time of aGvHD diagnosis has been linked with a severe disease phenotype (see Table 2 for details) [26].

### 4.2. SCFA-Producing Bacteria and Implications to Clinical Outcomes

SCFAs are key microbiota metabolites involved in the mechanisms through which the intestinal microbiota may influence clinical outcomes following allo-HSCT [25,26,28,29,30,42,47,48,49,50]. SCFAs, which include butyrate, propionate and acetate, play important roles in promoting gut homeostasis and regulating the immune system [42,47,48,49].

During allo-HSCT, there is extensive evidence demonstrating a decrease in SCFA levels and SCFA-producing bacteria (see Figure 1) [25,28,29,42]. In a study with 42 patients, fecal butyrate and propionate were measured at three timepoints: **(1)** Prior to allo-HSCT (baseline), **(2)** D + 7, and **(3)** D + 14 [25]. Compared to baseline, samples collected at D + 7 and D + 14 showed significantly decreased levels of both butyrate and propionate (*p*-values NR [Not Reported]) [25]. In another study involving 201 patients, stool samples were collected longitudinally at seven timepoints. These included **(1)** Prior to allo-HSCT, **(2)** D0, **(3)** D + 7, **(4)** D + 14, **(5)** D + 21, **(6)** D + 30, and **(7)** D + 90 [30]. This study demonstrated a strong and prolonged suppression of fecal butyrate levels. Significant reductions were observed from prior to allo-HSCT to D0 (*p* = 0.01; r = 0.5) and between prior to allo-HSCT and D + 7 (*p* = 0.003; r = 0.6) [30]. Similarly, in a study of 360 patients, SCFA-producing bacteria were assessed at the time of engraftment [29]. The majority of patients had either a low relative abundance (40.8%) or no detectable (40%) SCFA-producing bacteria, while only 19.2% had a high relative abundance [29]. These findings consistently outline a profound depletion of SCFA and their producers over the allo-HSCT journey, reinforcing their potential role in post-transplantation outcomes.

Indeed, previous studies have demonstrated that decreased levels of SCFAs and their producers contribute to poor allo-HSCT outcomes (see Table 3). Important clinical outcomes modulated by SCFA and their producers are: **(1)** Overall survival [28]; **(2)** GvHD [25,26,28,30,42]; **(3)** Transplantation-related mortality [30]; and **(4)** Viral lower respiratory tract infection [29]. For instance, in a study involving 360 patients, a high abundance of SCFA-producing bacteria at the engraftment period was independently associated with a fivefold decrease in the risk of viral lower respiratory tract infection (HR 0.22; 95% CI 0.04–0.69; *p* = 0.06) [29]. Furthermore, in a study of 64 allo-HSCT recipients, a higher abundance of *Blautia* (a key SCFA-producing genus) was independently associated with lower GvHD-related mortality (HR 0.18; 95% CI 0.05–0.63; *p* = 0.007) and reduced risk of refractory GvHD (HR 0.3; 95% CI 0.14–0.64; *p* = 0.002) [28]. These and other studies in the literature highlight the clinical relevance of preserving SCFA-producing bacteria over the allo-HSCT journey as a key strategy to improve patient outcomes.

### 4.3. Intestinal Domination and Implications to Clinical Outcomes

In patients undergoing allo-HSCT, another important microbiota fingerprint is the expansion of a single microbiota genus leading to intestinal domination. Intestinal domination is a frequent fingerprint that can occur in 28 to 65% of patients undergoing allo-HSCT [17,22,24,31,32,33,34,51]. Although intestinal domination is a prevalent fingerprint, the specific genus driving these events may vary across studies (see Supplementary Table S5). For example, in a multi-center study including 1325 patients, *Enterococcus* domination occurred in 65% of patients and was the most common genus to dominate the microbiota [32]. Similarly, *Enterococcus* was also the most common genus to dominate the microbiota in a study including 94 patients [34]. Nevertheless, in a study including 98 patients, *Streptococcus* was identified as the most common genus associated with domination events, occurring in 42% of patients [22]. In this study, other genera responsible for intestinal domination in increasing order were: **(1)** *Akkermansia* (28%), **(2)** *Blautia* (28%), **(3)** *Lactobacillus* (28%), **(4)** *Enterococcus* (36%), and **(5)** *Bacteroides* (38%) [22]. Overall, these findings suggest that while the dominant genus may vary across cohorts, *Enterococcus* consistently emerges as a key driver of intestinal domination events [17,22,24,31,32,33,34,51].

*Enterococcus* is not only a key driver of intestinal domination, but it is also the one most likely associated with poor outcomes. Indeed, while previous studies have linked *Enterococcus* domination with poor outcomes, domination by other genera does not appear to carry the same prognostic significance (see Table 4) [17,22,24,31,32,33,34,51]. For instance, in a study with 98 patients undergoing allo-HSCT, *Enterococcus* domination was associated with the following outcomes: bloodstream infection (63% vs. 35%; *p* = 0.01), *Clostridioides difficile* colitis (34% vs. 16%; *p* = 0.04), overall survival (*p* = 0.01), and treatment-related mortality (*p* = 0.02) [22]. In this same study, however, overall survival was not impacted by domination for the following genera: **(1)** *Bacteroides* (*p* = 0.08), **(2)** *Akkermansia* (*p* = 0.14), **(3)** *Blautia* (*p*-value NR), **(4)** *Lactobacillus* (*p* = 0.52), and **(5)** *Streptococcus* (*p* = 0.70) [22]. Other allo-HSCT studies have also highlighted the implications of *Enterococcus* domination. In a study including 1325 patients, *Enterococcus* domination was an independent risk factor for decreased overall survival (HR 2.06; 95% CI 1.50–2.82; *p* < 0.0001) [32]. *Enterococcus* domination was also associated with a nine-fold increase in the risk of bloodstream infections (HR 9.35; 95% CI 2.43–45.44; *p* = 0.001) in a study including 94 patients [34]. These studies suggest that *Enterococcus* domination is a key fingerprint with prognostic significance during allo-HSCT. Therefore, future studies should explore strategies to modulate *Enterococcus* domination.

## 5. Mechanistic Insights Modulating the Relationship Between Intestinal Microbiota and Allo-HSCT

While traditional microbiota studies have focused on taxonomic profiling and remain predominantly associative, the next frontier involves large-scale multiomics analysis to elucidate underlying mechanistic insights [52]. Results from these analyses have revealed that the intestinal microbiota influences host physiology through two primary mechanisms: **(1)** microbiota metabolites, and **(2)** direct receptor binding [53]. Microbiota metabolites, which range from small to large molecules, include SCFAs, indole, and secondary bile acids. Direct receptor binding between intestinal microbiota and human cells may occur through several mechanisms, including recognition of PAMPs (pathogen-associated molecular patterns) such as LPS (lipopolysaccharides) [53]. Given our special interest in SCFAs, the following paragraph will provide detailed data on how these metabolites may influence host physiology and allo-HSCT outcomes. Detailed information on the other mechanisms can be found in [53].

SCFAs are produced by the microbiota through the metabolism of non-digestible carbohydrates and regulate several metabolic pathways in the gut and distant organs (liver, adipose tissue, and brain) [53]. The main SCFAs include butyrate, propionate, and acetate [10,53]. SCFAs have key functions that influence immune response and intestinal homeostasis. First, SCFAs, especially butyrate, are an essential energy source for intestinal epithelial cells that contribute to maintaining the gut barrier integrity [10,53]. Also, SCFAs support goblet cells via upregulating mucin-related genes and preserve intestinal mucosal barrier [10,53]. Both functions may aid in preventing bacterial translocation [10]. SCFAs have also been linked to the control of the anaerobic environment in the colon through activation of β-oxidation in the mitochondria [53]. Furthermore, they act as a histone deacetylase inhibitor, facilitating the transcription of genes related to immune regulation and providing anti-inflammatory effects, with interleukin (IL)-10 secretion and regulatory T cell differentiation [10]. Taken together, these and other studies in the literature highlight the potential mechanisms by which SCFAs may impact the aforementioned allo-HSCT outcomes (see Section 4.2) [10,50,53,54]. Understanding these mechanistic insights is a key step towards the development of more efficient therapies.

## 6. Strategies to Modulate the Intestinal Microbiota During Allo-HSCT

Given the prognostic significance of microbiota fingerprints, several studies have explored strategies to modulate the intestinal microbiota during allo-HSCT [32,55,56]. Overall, two major strategies have been investigated: **(1)** fecal microbiota transplantation (FMT), and **(2)** dietary interventions [32,55,56,57,58,59]. These are promising strategies because they can modulate the complex relationship between microbiota and the immune system through mechanisms that control alloreactivity without further compromising the immune system [60]. These strategies have not only demonstrated the ability to restore microbiota diversity and key microbiota metrics, but have also been associated with better clinical outcomes during allo-HSCT [39,55].

FMT is a procedure in which stool from healthy donors (allogeneic FMT) or from the patient prior to dysbiosis (autologous FMT) is administered to restore intestinal microbiota balance [39,55]. In the allo-HSCT setting, FMT is an emerging therapy that has been shown to be feasible [55,60]. However, given the compromised immune system in patients undergoing allo-HSCT, safety concerns remain an important issue [61]. In general, the most common FMT adverse events reported by studies are mild and include diarrhea, bloating, nausea, vomiting, and abdominal pain [56,62,63,64]. However, severe adverse events may also occur and include sepsis, BSI, colitis, bowel perforation, hepatic encephalopathy and death [56,62,63,64]. Despite these concerns, studies are showing that severe adverse events, especially infections, are rare in the allo-HSCT setting [61]. Therefore, FMT has been used both as a prophylactic and therapeutic intervention for patients undergoing allo-HSCT [15,55].

Clinical outcomes that have been improved by FMT include: **(1)** GvHD [60,65], **(2)** Drug-resistant bacteria colonization [65,66,67,68], **(3)** BSI [68], **(4)** mortality [67], and **(5)** *Clostridioides difficile* colitis [69,70,71]. For instance, in a study involving 15 adult patients with steroid-refractory or steroid-dependent GvHD that received allogeneic FMT, 66.7% achieved a complete clinical response (resolution of GvHD symptoms) within one month after treatment [60]. Furthermore, patients who responded to the FMT exhibited a significant increase in both intestinal diversity and the abundance of SCFA-producing bacteria [60]. Another study examined 19 adult patients colonized with multidrug-resistant organisms, of whom 8 received FMT [72]. Compared to those who did not receive FMT, patients treated with FMT demonstrated significantly higher 12-month overall survival (70% vs. 36%; *p* = 0.044) and required fewer intensive care admissions (0% vs. 46%; *p* = 0.045) [72]. Although these studies show promising data, significant challenges remain before FMT can become a standardized treatment in the allo-HSCT setting [61,73]. The available literature demonstrates heterogeneity in FMT approaches, including differences in manufacturing process, route of administration, timing, dosing strategies and donor selection. Additionally, significant regulatory challenges persist regarding FMT [73,74]. Regulatory bodies lack consensus on the classification and regulation of FMT, with some countries categorizing it as a drug, while others classify it as a medicinal product or tissue [73,74]. In addition, several countries have yet to establish any regulatory framework for FMT [74]. This lack of harmonization across all aspects of FMT research poses significant barriers to clinical trial development and clinical translation. Although current preliminary findings are encouraging, further large-scale randomized clinical trials employing standardized methodologies are needed.

Dietary interventions represent another promising therapeutic strategy, encompassing **(1)** Prebiotics, **(2)** Probiotics, **(3)** Synbiotics, and **(4)** Route of nutritional support [32,58,59,75,76,77,78]. These dietary interventions have been mostly associated with improvement in the following allo-HSCT clinical outcomes: **(1)** GvHD [32,57,58,59], **(2)** Diarrhea [77], **(3)** Mortality [59,75,77], and **(4)** Mucositis [77]. In a pilot randomized clinical trial including 40 adult patients, 20 patients received daily synbiotics (seven bacterial strains + fructo-oligosaccharides) [58]. When compared to the control group, patients receiving synbiotics had lower rates of severe GvHD (0% vs. 25%; *p* = 0.047) [58]. Similarly, in another study including 44 adult patients, 22 patients received GFO (combination of glutamine, fiber and oligosaccharides) [77]. Patients receiving GFO had a statistically significant reduction in diarrhea duration (3.73 vs. 7.68 days; *p* < 0.0001) and mucositis duration (3.86 vs. 6.00 days; *p* < 0.0330). GFO administration was also associated with higher survival rate at 100 days after allo-HSCT (100% vs. 77.3%; *p* = 0.0091). Nevertheless, it is important to note that most available studies are observational with small sample sizes. Furthermore, studies demonstrate significant heterogeneity across multiple parameters, including substance dosage, administration timing and routes, and strain selection. Therefore, future research employing larger sample sizes and standardized methodologies is warranted to strengthen the evidence base.

## 7. Challenges in Translating Intestinal Microbiota Research into Allo-HSCT Clinical Practice

Although significant advances have been made in elucidating the role of intestinal microbiota in the allo-HSCT context, microbiota research remains distant from clinical implementation [79,80]. This loss of translation between microbiota research and allo-HSCT clinical practice stems from several factors, including biological, methodological and logistical challenges [79,80].

The biological challenges are fundamentally rooted in the paucity of evidence supporting mechanistic hypotheses that causally link intestinal microbiota alterations to allo-HSCT outcomes [79,80]. Most available literature remains associative and, thus, it remains unclear whether intestinal microbiota changes represent a cause or consequence of clinical outcomes during the allo-HSCT process [79]. For instance, the presence of the aforementioned fingerprints could be the consequence of several allo-HSCT variables, such as severe underlying disease, multiple hospitalizations, infectious complications and antibiotic use. Methodological challenges arise primarily from the substantial inter-study variability regarding protocols for intestinal microbiota analysis. Studies exhibit substantial variability across multiple parameters, including sample collection techniques, collection timing, DNA sequencing methodologies and bioinformatics pipelines [79]. Furthermore, the effects of confounding variables that may influence the intestinal microbiota are not consistently accounted for, such as dietary patterns, environmental factors and concurrent medications [79]. Additionally, while microbiota research endorses personalized therapeutic strategies, the logistical challenges associated with implementing microbiota profiling and patient-tailored treatment protocols present substantial barriers to clinical translation [79]. Finally, microbiota studies are often single-center studies that include small to modest cohorts, which hampers the generalizability of the findings [79]. In summary, the substantial variability in microbiota research and lack of mechanistic studies, coupled with the inherent complexity of microbiome analyses and inadequate control of confounding variables (diet, antibiotic use, geographic distribution), prevent most clinicians from integrating microbiota research into allo-HSCT clinical practice [79,80].

**Figure 3:**
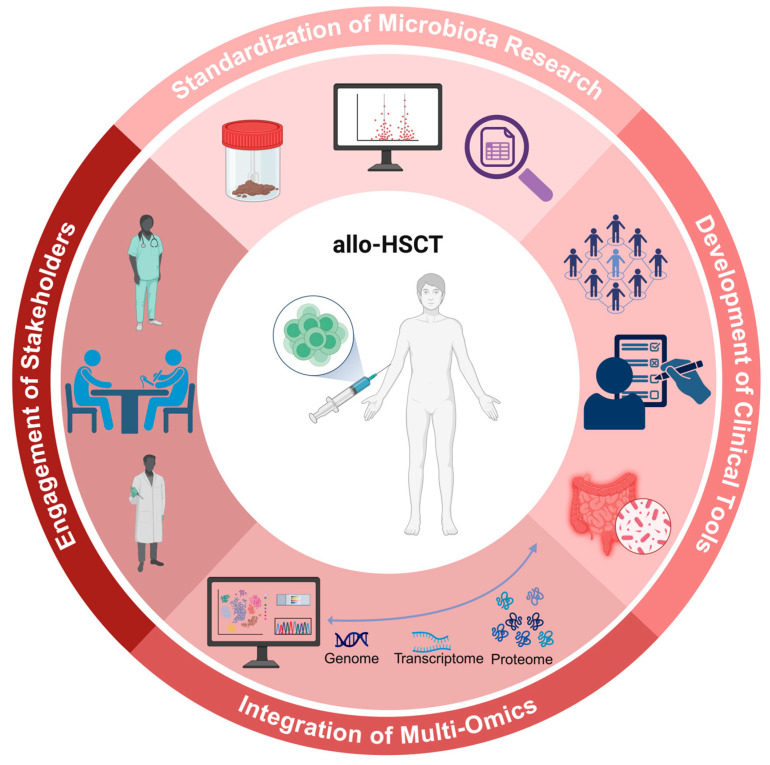
Future directions of intestinal microbiota research in the allo-HSCT context: (1) Standardization of microbiota research, (2) Development of clinical tools, (3) Integration of multi-omics, and (4) Engagement of Stakeholders. Allo-HSCT = allogeneic hematopoietic stem cell transplantation. Created with BioRender.com, Soares Ferreira Junior A. (2025) https://BioRender.com/5d47vvk (accessed on 19 September 2025).

To overcome the aforementioned challenges, a multifaceted strategy is required (see Figure 3) [79]. The most critical intervention may be the standardization of microbiota research, which is currently advancing through initiatives such as the STORMS checklist (“Strengthening The Organization and Reporting of Microbiome Studies”), FDA (Food and Drug Administration) oversight and consensus guidelines [73,80,81]. This standardization should ideally encompass all phases of microbiota research, spanning from initial test indication to the reporting and clinical interpretation of microbiota findings [80]. Furthermore, while traditional microbiota studies have focused primarily on taxonomic profiling, future multi-omics studies may provide deeper insights [52]. Host-microbiota interactions and their underlying mechanisms could be better understood through these multi-omics studies, which correlate genomic, transcriptomic, proteomic, and metabolomic data. Additionally, interventional studies should comply with FDA regulations when assessing the efficacy and safety of fecal microbiota transplantation products [73]. Equally important is addressing logistical challenges and designing microbiota studies that answer relevant clinical questions and provide outputs applicable to clinical practice [79,80]. To this end, studies should: **(1)** actively engage key stakeholders—including patients, clinicians, scientists and industry partners—in the research process, and **(2)** conduct rigorous sample size estimation to enhance reliability and generalizability [79,80]. Additionally, researchers should prioritize clinical outcomes with direct relevance to clinical practice [79]. Furthermore, researchers should aim to translate complex microbiota findings into accessible clinical tools that assist physicians with patient stratification and prognosis (risk scores). Finally, fostering communication between microbiota scientists and the medical community through targeted educational initiatives and translational grant opportunities will equip physicians with the requisite knowledge to integrate microbiota research into clinical practice [79].

## 8. Conclusions and Future Directions

This review identified three key intestinal microbiota fingerprints associated with allo-HSCT outcomes: decreased intestinal microbiota diversity, reduced abundance of SCFA-producing bacteria, and *Enterococcus* intestinal domination. Although intestinal microbiota represents a key prognostic factor and therapeutic target in patients undergoing allo-HSCT, further translation of this knowledge into clinical practice is needed. Future large-scale clinical studies with standardized microbiota methodologies and mechanistic evaluation should be designed in collaboration with key stakeholders, including physicians, microbiome scientists, and patients. Such collaborative approaches will enhance study reliability, generalizability, and assessment of outcomes directly relevant to clinical practice.

## Figures and Tables

**Table 1:** Implications of Intestinal Diversity Prior to allo-HSCT.

Outcomes	Author, Year N	Finding
**Overall Survival** [16,17,21]	Peled 2020 [17] 606	↓ Overall Mortality Higher alpha diversity prior to allo-HSCT was associated with a lower risk of mortality (HR 0.41; 95% CI 0.24–0.71)
	Liu 2017 [16] 57	↓ Overall Mortality Patients with higher phylogenetic diversity had lower overall mortality rates (HR 0.37; 95% CI 0.18–0.77; *p* = 0.008)
	Masetti 2023 [21] ^β^ 90	↑ Overall Survival Patients with higher intestinal diversity exhibited a higher probability of overall survival (88.9% ± 5.7% vs. 62.7% ± 8.2%; *p* = 0.011).
**Transplantation-related mortality** [17]	Peled 2020 [17] 606	↓ Transplant-related mortality Higher alpha diversity prior to allo-HSCT was associated with a lower risk of transplant-related mortality (HR 0.44; 95% CI 0.22–0.87).
**aGvHD** [21]	Masetti 2023 [21] ^β^ 90	↓ aGvHD The cumulative incidence of grade 2 to 4 aGvHD was significantly lower in the higher diversity group than in the lower diversity group (20.0% ± 6.0% [SE] vs. 44.4% ± 7.4% [SE]; *p* = 0.017). The cumulative incidence of grade 3 to 4 aGvHD was significantly lower in the higher diversity group than in the lower diversity group (2.2% ± 2.2% [SE] vs. 20.0% ± 6.0% [SE]; *p* = 0.007).
	Biagi 2019 [35] 36	The diversity between pre-HSCT samples were greater in individuals who developed intestinal GvHD (0.86 ± 0.15) than in individuals without GvHD (0.72 ± 0.15, *p* = 0.001) and individuals who developed less severe skin GvHD (0.77 ± 0.15, *p* = 0.02).

Allo-HSCT = Allogeneic hematopoieitic stem cell transplantation; CI = Confidence interval; aGvHD = acute graft versus host disease; HR = Hazard ratio; N = number of patients included in this analysis; SE = Standard error; ↓ = decreased; ↑ = increased; ^β^ = included only pediatric patients.

**Table 2:** Clinical Implications of Intestinal Diversity Over The allo-HSCT.

Outcome	Author, Year N Sample Timing	Finding
**Overall Survival** [17,23,24]	Peled 2020 [17] 704 At engraftment	↑ Overall Survival Patients were categorized into low- vs. high-diversity groups based on the median value. High diversity at engraftment was associated with a significant improve in overall survival (HR 0.75; 95% CI 0.58–0.96). This association was also identified after multivariable adjustment for age, intensity of the conditioning regimen, graft source and HCT-CI (HR 0.71; 95% CI 0.55–0.92). When considered as a continuous variable, high intestinal diversity was also associated with improved overall survival in both univariate (HR 0.58; 95% CI 0.37–0.91) and multivariate (HR 0.50; 95% CI 0.31–0.80) analysis.
	Taur 2014 [23] 80 At engraftment	↑ Overall Survival Overall survival at 3 years was 36%, 60% and 67% for low, intermediate and high diversity groups (*p* = 0.19). Patients with low diversity (inverse Simpson < 2) were 3 times more likely to die within the follow-up when compared to those with higher microbial diversity (HR 3.13, 95% CI 1.39–7.98; *p* = 0.05; adjusted HR 2.56; 95% CI 1.03–7.23; *p* = 0.42). Low diversity showed a strong effect on mortality after multivariate adjustment for other clinical predictors (transplant related mortality: adjusted hazard ratio, 5.25; *p* = 0.014).
	Gu 2022 [24] 86 At engraftment	↑ Overall Survival Patients were categorized into low- vs. high- diversity groups based on the median Shannon Index value. When compared to patients with low diversity, patients with high diversity had significantly higher two-year overall survival (83.7% vs. 60.6%; *p* = 0.026). After adjusting for disease risk, pretransplant comorbidity, and previous chemotherapy, low intestinal diversity was an independent predictor of all-cause death (HR 2.62; 95% CI 1.06–6.49; *p* = 0.038) in a multivariate analysis.
**Transplantation-related mortality** [23,24,41]	Taur 2014 [23] 80 At engraftment	↑ Transplant-related mortality Transplant-related mortality was 9%, 23%, and 53% for high, intermediate and low diversity groups, respectively (*p* = 0.03). Patients with low diversity (inverse Simpson < 2) were 7.5 times more likely to experience transplant-related mortality within the follow-up when compared to those with higher microbial diversity (HR 7.54; 95% CI 2.12–47.88; *p* = 0.001; adjusted hazard ratio, 5.25; 95% CI 1.36–35.07; *p* = 0.014).
	Gu 2022 [24] 86 At engraftment	↑ Transplantation-related Mortality When compared to patients in the high diversity group, patients in the low-diversity group had higher estimated 2-year transplanted related mortality (20.0% vs. 4.7%; *p* = 0.04). After adjusting for pretransplant comorbidity, disease status at the time of allo-HSCT and previous chemotherapy, low intestinal diversity was an independent predictor of transplant-related mortality (HR 4.95; 95% CI 1.03–23.76; *p* = 0.046).
	Galloway-Pena 2019 [41] 44 At engraftment	↓ Transplantation-related Mortality The Shannon diversity index at the time of engraftment was significantly associated with transplantation-related mortality (coefficient = −1.44; *p* = 0.02)
**aGvHD** [20,23,26,28,41,46]	Jenq 2015 [28] 64 D + 12	↓ GvHD-related mortality Increased intestinal diversity was associated with reduced GvHD-related mortality (*p* = 0.005).
	Mancini 2017 [20] 96 D + 10	↑ aGvHD Decreased intestinal diversity at D + 10 was associated with increased risk of early onset aGvHD (OR 7.833; 95% CI 2.141–28.658; *p* = 0.038).
	Taur 2014 [23] 80 At engraftment	↑ GvHD-related mortality GvHD-related mortality was higher in patients with low diversity (*p* = 0.018).
	Payen 2020 [26] 70 At the onset of GvHD	↑ aGvHD severity Patients with severe aGvHD had significantly lower indexes of alpha diversity: Chao1 (*p* = 0.039) and Simpson (*p* = 0.013)
	Golob 2017 [46] 66 At engraftment Weekly samples from prior to allo-HSCT until D + 100	↑ aGvHD severity Patients with severe aGvHD had a significantly lower alpha diversity index compared to both the control group and patients without severe aGvHD (*p* < 0.05). This finding was statistically significant when analyzing all stool samples collected over the allo-HSCT and when analyzing only samples collected at the engraftment period.
	Galloway-Pena 2019 [41] 44 At engraftment	The Shannon diversity index at the time of engraftment was significantly associated with the incidence of aGvHD (*p* = 0.02)
**Infections** [23]	Taur 2014 [23] 80 At engraftment	↑ Infection-related mortality Infection related mortality was higher in patients with low diversity (*p* = 0.018).

Allo-HSCT = allogeneic hematopoietic stem cell transplantation; N = number of patients included in the analysis; aGvHD = acute graft versus host disease; ↓ = decreased; ↑ = increased.

**Table 3:** Implications of SCFA-producing Bacteria and SCFA Levels Over allo-HSCT.

Outcomes	Author, Year N Sample Timing	Finding
**Overall Survival** [28]	Jenq 2015 [28] 64 D + 12	↑ Overall Survival Increased *Blautia* abundance was strongly associated with improved overall survival (*p* < 0.001).
**Transplantation-related Mortality** [30]	Meedt, 2022 [30] 201 aGvHD onset//D + 30	↑ Transplant-related Mortality Low BCoAT copy numbers at D + 30/GvHD were significantly associated with increased risk of transplant-related mortality (HR 4.459; 95% CI 1.1018–19.530; *p* = 0.047).
**aGvHD** [25,26,28,30,42]	Jenq 2015 [28] 64 D + 12	↓ GvHD-related mortality By using a taxonomic discovery analysis, increase in the genus *Blautia* was significantly associated with reduced GvHD-related mortality (*p* = 0.01). By stratifying patients based on *Blautia* median abundance, patients with higher abundance had reduced GvHD-related mortality (*p* = 0.04). In a multivariable analysis, *Blautia* abundance remained associated with GvHD-related mortality (HR 0.18; 95% CI 0.05–0.63; *p* = 0.007). ↓ Refractory GvHD Increased *Blautia* abundance was associated with reduced development of acute GvHD that required treatment with systemic corticosteroids or was steroid refractory (*p* = 0.01). In a multivariable analysis, *Blautia* abundance remained associated with refractory GvHD (HR 0.3; 95% CI 0.14–0.64; *p* = 0.002). ↓ Liver GvHD Increased *Blautia* abundance was associated with reduced liver GvHD (*p* = 0.02).
	Payen 2020 [26] 70 aGvHD onset	↑ aGvHD severity When compared to controls (patients undergoing allo-HSCT without GvHD), patients with severe GvHD had a significant depletion of the *Blautia coccoides group* (*p* = 0.07). Similar findings were found when compared to patients with mild aGvHD (*p* = 0.036). ↑ aGvHD severity When compared to controls (patients undergoing allo-HSCT without GvHD), patients with severe GvHD had a significant depletion of *Anaerostipes* (*p* = 0.015). ↑ aGvHD severity When compared to controls (patients undergoing allo-HSCT without GvHD), patients with severe GvHD had a significant depletion of *Faecalibacterium* (*p* = 0.011). ↑ aGvHD severity When compared to controls (patients undergoing allo-HSCT without GvHD), patients with severe GvHD had a significant depletion of *Lachnoclostridium* (*p* = 0.019). ↑ GvHD severity When compared to controls (patients undergoing allo-HSCT without GvHD), patients with severe GvHD had significantly lower levels of total SCFAs (12.50 vs. 2.42; *p* = 0.0003), acetate (8.87 vs. 2.15; *p* = 0.002), butyrate (1.11 vs. 0.06; *p* = 0.001), and propionate (2.33 vs. 0.10; *p* = 0.0009).
	Romick-Rosendale 2018 [25] 42 D + 14	↓ GvHD When compared to patients that developed GvHD, patients without GvHD had significantly higher levels of butyrate (1.77 vs. 0.0550; *p* = 0.0142), propionate (6.63 vs. 0.208; *p* = 0.0108) and acetate (39.6 vs. 7.92; *p* = 0.047) at samples collected at D + 14.
	Meedt, 2022 [30] 201 aGvHD onset//D + 30	↑ GI-GvHD severity Low BCoAT copy numbers at GvHD onset were correlated with GI-GvHD severity (*p* = 002; *r* = 0.3). ↑ GI-GvHD Patients with GI-GvHD had lower BCoAT copy numbers than patients with other organs manifestations (0 copies vs. 3.16 × 10^6^ copies; *p* = 0.006; *r* = 0.3). ↑ GvHD-related Mortality Patients with low BCoAT copy numbers displayed significantly higher GvHD-associated mortality rate than those with high BCoAT concentrations (*p* = 0.04).
	Artacho 2024 [42] 70 Prior to allo-HSCT and Engraftment	↑ GvHD A significant decrease in acetate levels was detected in patients who developed GvHD (log2FC median = −2.36; *p* = 0.049).
**Infections** [29]	Haak 2018 [29] 360 At engraftment	↓ LRTI The incidence of viral LRTI at 180 days was 17.3% and 16.1% for groups in which butyrate-producing bacteria were absent or low, respectively, and 3.2% for the high butyrate-producing group (*p* = 0.005). Patients with the highest abundance of butyrate-producing bacteria were independently associated with a fivefold decrease in risk of viral LRTI (HR 0.22; 95% CI 0.04–0.69; *p* = 0.06).

Allo-HSCT = allogeneic hematopoietic stem cell transplantation; BCoAT = Butyryl-CoA/Acetate CoA-Transferase Gene Copy; CI = Confidence interval; D = day; LRTI = Lower respiratory tract infection; GvHD = graft versus host disease; SCFA = Short chain fatty acid; HR = Hazard ratio; N = number of patients included in the analysis; ↓ = decreased; ↑ = increased.

**Table 4:** Implications of Intestinal Domination to Clinical Outcomes.

Outcomes	Autor, Year N Sample Timing	Implication
**Overall Survival** [22,32]	Messina 2024 [22] 98 Stools were collected once prior to HSCT, weekly until D + 30 and then at days D + 45, D + 90 and D + 180	↓ Overall survival Patients with *Enterococcus* domination had decreased overall survival (*p* = 0.01). Overall survival *Bacteroides* domination at any time point was not significantly associated with overall survival (*p* = 0.08). *Akkermansia* domination at any time point was not significantly associated with overall survival (*p* = 0.14). *Blautia* domination at any time point was not significantly associated with overall survival (*p* value NR). *Lactobacillus* domination was not significantly associated with overall survival (*p* = 0.52). *Streptococcus* domination was not significantly associated with overall survival (*p* = 0.70).
	Stein-Thoeringer 2019 [32] 1325 Samples were collected in the early post-transplant period (D0 to D + 21)	↓ Overall survival Patients with *Enterococcus* domination in the early-post transplant period had significantly reduced overall survival in univariate analysis (HR 1.97; 95% CI 1.45–2.66; *p* < 0.001). This finding remained significant in a multivariate analysis adjusted for graft source, age, conditioning intensity, gender and underlying disease (HR 2.06; 95% CI 1.50–2.82; *p* < 0.0001).
**Transplantation-related Mortality** [22]	Messina 2024 [22] 98 Stools were collected once prior to HSCT, weekly until D + 30 and then at days D + 45, D + 90 and D + 180	↑ Treatment-related mortality Patients with *Enterococcus* domination had increased treatment-related mortality (*p* = 0.02).
**aGvHD** [32]	Stein-Thoeringer 2019 [32] 1325 Samples were collected in the early post-transplant period (D0 to D + 21)	↑ GvHD-related mortality Patients with *Enterococcus* domination in the early-post transplant period had significantly increased GvHD-related mortality in univariate analysis (HR 2.04; 95% CI 1.18–3.52; *p* = 0.05). This finding remaining significant in a multivariate analysis adjusted for graft source, age, conditioning intensity, gender and underlying disease (HR 2.60; 95% CI 1.46–4.62; *p* < 0.01). ↑ GvHD severity (grade 2–4) Patients with *Enterococcus* domination in the early-post transplant period had significantly increased GvHD severity (grade 2–4) in univariate analysis (HR 1.44; 95% CI 1.10–1.88; *p* < 0.01). This finding remained significant in a multivariate analysis adjusted for graft source, age, conditioning intensity, gender and underlying disease (HR 1.32; 95% CI 1.00–1.75; *p* < 0.05).
**Infections** [22,34]	Messina 2024 [22] 98 Stools were collected once prior to HSCT, weekly until D + 30 and then at days D + 45, D + 90 and D + 180	↑ BSI Patients with *Enterococcus* domination at any time point had increased risk for BSI (63% vs. 35%; *p* = 0.01).
	Taur 2012 [34] 94 Prior to allo-HSCT After allo-HSCT (until D + 35)	↑ BSI Patients with *Enterococcus* domination had a 9-fold increased risk of VRE bacteremia (HR 9.35; 95% CI 2.43–45.44; *p* = 0.001).
	Taur 2012 [34] 94 Prior to allo-HSCT After allo-HSCT (until D + 35)	↑ BSI Patients with Proteobacteria domination had a 5-fold increased risk of Gram-negative bacteremia (HR 5.46; 95% CI 1.03–19.91; *p* = 0.047).
***Clostridioides difficile* colitis** [22]	Messina 2024 [22] 98 Stools were collected once prior to HSCT, weekly until D + 30 and then at days D + 45, D + 90 and D + 180	↑ *Clostridioides difficile* colitis Patients with *Enterococcus* domination at any time point had increased risk for BSI (34% vs. 16%; *p* = 0.04).
**Other** [22]	Messina 2024 [22] 98 Stools were collected once prior to HSCT, weekly until D + 30 and then at days D + 45, D + 90 and D + 180	↑ Relapse-related mortality Patients with *Enterococcus* domination had increased relapse-related mortality (*p* = 0.08).

Allo-HSCT = allogeneic hematopoietic stem cell transplantation; BSI = Bloodstream infection; CI = Confidence interval; D = day; GvHD = graft versus host disease; N = Number of patients included in this analysis; HR = Hazard ratio; ↓ = decreased; ↑ = increased.

## Data Availability

No new data were created or analyzed in this study.

## Supplementary Materials

The following supporting information can be downloaded at: https://www.mdpi.com/article/10.3390/jcm14196874/s1, Table S1: Key Studies Evaluating the Intestinal Microbiota Prior to allo-HSCT; Table S2: Implications of Intestinal Microbiota Fingerprints Prior to Allo-HSCT; Table S3: Key Studies Evaluating the Dynamics of Intestinal Diversity Over the Allo-HSCT; Table S4: Key Studies Evaluating the Dynamics of SCFA-producing Bacteria and SCFA Levels Over the Allo-HSCT; Table S5: Dynamics of Intestinal Domination Over Allo-HSCT. References [14,16,17,19,20,21,22,23,24,25,27,28,29,30,31,32,33,34,35,36,41,42] are cited in Supplementary Materials.

## Abbreviations

The following abbreviations are used in this manuscript:

Allo-HSCT	Allogeneic hematopoietic stem cell transplantation
BSI	Bloodstream infection
CI	Confidence interval
CMV	Cytomegalovirus
D	Day
FDA	Food and Drug Administration
FMT	Fecal microbiota transplantation
NR	Not Reported
LRTI	Lower respiratory tract infection
SCFA	Short-chain fatty acid
SE	Standard error
STORMS	Strengthening The Organization and Reporting of Microbiome Studies
GvHD	Graft versus host disease
GFO	Glutamine, fiber and oligosaccharides
HR	Hazard Ratio
HLA	Human Leukocyte Antigen
TRM	Transplantation-related mortality

## References

[1] Hill G.R., Betts B.C., Tkachev V., Kean L.S., Blazar B.R. (2021). Current Concepts and Advances in Graft-Versus-Host Disease Immunology. Annu. Rev. Immunol..

[2] Ferrara J.L., Levine J.E., Reddy P., Holler E. (2009). Graft-versus-Host Disease. Lancet.

[3] Jagasia M., Arora M., Flowers M.E.D., Chao N.J., McCarthy P.L., Cutler C.S., Urbano-Ispizua A., Pavletic S.Z., Haagenson M.D., Zhang M.-J. (2012). Risk Factors for Acute GVHD and Survival after Hematopoietic Cell Transplantation. Blood.

[4] Ilett E.E., Jørgensen M., Noguera-Julian M., Nørgaard J.C., Daugaard G., Helleberg M., Paredes R., Murray D.D., Lundgren J., MacPherson C. (2020). Associations of the Gut Microbiome and Clinical Factors with Acute GVHD in Allogeneic HSCT Recipients. Blood Adv..

[5] Nesher L., Rolston K.V.I., Safdar A. (2019). Febrile Neutropenia in Transplant Recipients. Principles and Practice of Transplant Infectious Diseases.

[6] Barrett A.J., Battiwalla M. (2010). Relapse after Allogeneic Stem Cell Transplantation. Expert. Rev. Hematol..

[7] Horowitz M., Schreiber H., Elder A., Heidenreich O., Vormoor J., Toffalori C., Vago L., Kröger N. (2018). Epidemiology and Biology of Relapse after Stem Cell Transplantation. Bone Marrow Transpl..

[8] Lynch S.V., Pedersen O. (2016). The Human Intestinal Microbiome in Health and Disease. N. Engl. J. Med..

[9] Malard F., Holler E., Sandmaier B.M., Huang H., Mohty M. (2023). Acute Graft-versus-Host Disease. Nat. Rev. Dis. Primers.

[10] Fujiwara H. (2021). Crosstalk Between Intestinal Microbiota Derived Metabolites and Tissues in Allogeneic Hematopoietic Cell Transplantation. Front. Immunol..

[11] Nobels A., Van Marcke C., Jordan B.F., Van Hul M., Cani P.D. (2025). The Gut Microbiome and Cancer: From Tumorigenesis to Therapy. Nat. Metab..

[12] Rashidi A., Kaiser T., Shields-Cutler R., Graiziger C., Holtan S.G., Rehman T.U., Wasko J., Weisdorf D.J., Dunny G., Khoruts A. (2019). Dysbiosis Patterns during Re-Induction/Salvage versus Induction Chemotherapy for Acute Leukemia. Sci. Rep..

[13] Galloway-Peña J.R., Smith D.P., Sahasrabhojane P., Ajami N.J., Wadsworth W.D., Daver N.G., Chemaly R.F., Marsh L., Ghantoji S.S., Pemmaraju N. (2016). The Role of the Gastrointestinal Microbiome in Infectious Complications during Induction Chemotherapy for Acute Myeloid Leukemia. Cancer.

[14] Kusakabe S., Fukushima K., Maeda T., Motooka D., Nakamura S., Fujita J., Yokota T., Shibayama H., Oritani K., Kanakura Y. (2020). Pre- and Post-serial Metagenomic Analysis of Gut Microbiota as a Prognostic Factor in Patients Undergoing Haematopoietic Stem Cell Transplantation. Br. J. Haematol..

[15] Henig I., Yehudai-Ofir D., Zuckerman T. (2020). The Clinical Role of the Gut Microbiome and Fecal Microbiota Transplantation in Allogeneic Stem Cell Transplantation. Haematologica.

[16] Liu C., Frank D.N., Horch M., Chau S., Ir D., Horch E.A., Tretina K., Van Besien K., Lozupone C.A., Nguyen V.H. (2017). Associations between Acute Gastrointestinal GvHD and the Baseline Gut Microbiota of Allogeneic Hematopoietic Stem Cell Transplant Recipients and Donors. Bone Marrow Transpl..

[17] Peled J.U., Gomes A.L.C., Devlin S.M., Littmann E.R., Taur Y., Sung A.D., Weber D., Hashimoto D., Slingerland A.E., Slingerland J.B. (2020). Microbiota as Predictor of Mortality in Allogeneic Hematopoietic-Cell Transplantation. N. Engl. J. Med..

[18] Zhou Y., Zhou C., Zhang A. (2022). Gut Microbiota in Acute Leukemia: Current Evidence and Future Directions. Front. Microbiol..

[19] Holler E., Butzhammer P., Schmid K., Hundsrucker C., Koestler J., Peter K., Zhu W., Sporrer D., Hehlgans T., Kreutz M. (2014). Metagenomic Analysis of the Stool Microbiome in Patients Receiving Allogeneic Stem Cell Transplantation: Loss of Diversity Is Associated with Use of Systemic Antibiotics and More Pronounced in Gastrointestinal Graft-versus-Host Disease. Biol. Blood Marrow Transplant..

[20] Mancini N., Greco R., Pasciuta R., Barbanti M.C., Pini G., Morrow O.B., Morelli M., Vago L., Clementi N., Giglio F. (2017). Enteric Microbiome Markers as Early Predictors of Clinical Outcome in Allogeneic Hematopoietic Stem Cell Transplant: Results of a Prospective Study in Adult Patients. Open Forum Infect. Dis..

[21] Masetti R., Leardini D., Muratore E., Fabbrini M., D’Amico F., Zama D., Baccelli F., Gottardi F., Belotti T., Ussowicz M. (2023). Gut Microbiota Diversity before Allogeneic Hematopoietic Stem Cell Transplantation as a Predictor of Mortality in Children. Blood.

[22] Messina J.A., Tan C.Y., Ren Y., Hill L., Bush A., Lew M., Andermann T., Peled J.U., Gomes A., Van Den Brink M.R.M. (2024). *Enterococcus* Intestinal Domination Is Associated with Increased Mortality in the Acute Leukemia Chemotherapy Population. Clin. Infect. Dis..

[23] Taur Y., Jenq R.R., Perales M.-A., Littmann E.R., Morjaria S., Ling L., No D., Gobourne A., Viale A., Dahi P.B. (2014). The Effects of Intestinal Tract Bacterial Diversity on Mortality Following Allogeneic Hematopoietic Stem Cell Transplantation. Blood.

[24] Gu Z., Xiong Q., Wang L., Wang L., Li F., Hou C., Dou L., Zhu B., Liu D. (2022). The Impact of Intestinal Microbiota in Antithymocyte Globulin–Based Myeloablative Allogeneic Hematopoietic Cell Transplantation. Cancer.

[25] Romick-Rosendale L.E., Haslam D.B., Lane A., Denson L., Lake K., Wilkey A., Watanabe M., Bauer S., Litts B., Luebbering N. (2018). Antibiotic Exposure and Reduced Short Chain Fatty Acid Production after Hematopoietic Stem Cell Transplant. Biol. Blood Marrow Transplant..

[26] Payen M., Nicolis I., Robin M., Michonneau D., Delannoye J., Mayeur C., Kapel N., Berçot B., Butel M.-J., Le Goff J. (2020). Functional and Phylogenetic Alterations in Gut Microbiome Are Linked to Graft-versus-Host Disease Severity. Blood Adv..

[27] Sardzikova S., Andrijkova K., Svec P., Beke G., Klucar L., Minarik G., Bielik V., Kolenova A., Soltys K. (2024). Gut Diversity and the Resistome as Biomarkers of Febrile Neutropenia Outcome in Paediatric Oncology Patients Undergoing Hematopoietic Stem Cell Transplantation. Sci. Rep..

[28] Jenq R.R., Taur Y., Devlin S.M., Ponce D.M., Goldberg J.D., Ahr K.F., Littmann E.R., Ling L., Gobourne A.C., Miller L.C. (2015). Intestinal Blautia Is Associated with Reduced Death from Graft-versus-Host Disease. Biol. Blood Marrow Transplant..

[29] Haak B.W., Littmann E.R., Chaubard J.-L., Pickard A.J., Fontana E., Adhi F., Gyaltshen Y., Ling L., Morjaria S.M., Peled J.U. (2018). Impact of Gut Colonization with Butyrate Producing Microbiota on Respiratory Viral Infection Following Allo-HCT. Blood.

[30] Meedt E., Hiergeist A., Gessner A., Dettmer K., Liebisch G., Ghimire S., Poeck H., Edinger M., Wolff D., Herr W. (2022). Prolonged Suppression of Butyrate-Producing Bacteria Is Associated with Acute Gastrointestinal Graft-vs-Host Disease and Transplantation-Related Mortality After Allogeneic Stem Cell Transplantation. Clin. Infect. Dis..

[31] Chhabra S., Szabo A., Clurman A., McShane K., Waters N., Eastwood D., Samanas L., Fei T., Armijo G., Abedin S. (2022). Mitigation of Gastrointestinal Graft-*versus*-Host Disease with Tocilizumab Prophylaxis Is Accompanied by Preservation of Microbial Diversity and Attenuation of Enterococcal Domination. Haematologica.

[32] Stein-Thoeringer C.K., Nichols K.B., Lazrak A., Docampo M.D., Slingerland A.E., Slingerland J.B., Clurman A.G., Armijo G., Gomes A.L.C., Shono Y. (2019). Lactose Drives *Enterococcus* Expansion to Promote Graft-versus-Host Disease. Science.

[33] Fujimoto K., Hayashi T., Yamamoto M., Sato N., Shimohigoshi M., Miyaoka D., Yokota C., Watanabe M., Hisaki Y., Kamei Y. (2024). An Enterococcal Phage-Derived Enzyme Suppresses Graft-versus-Host Disease. Nature.

[34] Taur Y., Xavier J.B., Lipuma L., Ubeda C., Goldberg J., Gobourne A., Lee Y.J., Dubin K.A., Socci N.D., Viale A. (2012). Intestinal Domination and the Risk of Bacteremia in Patients Undergoing Allogeneic Hematopoietic Stem Cell Transplantation. Clin. Infect. Dis..

[35] Biagi E., Zama D., Rampelli S., Turroni S., Brigidi P., Consolandi C., Severgnini M., Picotti E., Gasperini P., Merli P. (2019). Early Gut Microbiota Signature of aGvHD in Children given Allogeneic Hematopoietic Cell Transplantation for Hematological Disorders. BMC Med. Genom..

[36] Doki N., Suyama M., Sasajima S., Ota J., Igarashi A., Mimura I., Morita H., Fujioka Y., Sugiyama D., Nishikawa H. (2017). Clinical Impact of Pre-Transplant Gut Microbial Diversity on Outcomes of Allogeneic Hematopoietic Stem Cell Transplantation. Ann. Hematol..

[37] Luo Y., Sheikh T.M.M., Li X., Yuan Y., Yao F., Wang M., Guo X., Wu J., Shafiq M., Xie Q. (2024). Exploring the Dynamics of Gut Microbiota, Antibiotic Resistance, and Chemotherapy Impact in Acute Leukemia Patients: A Comprehensive Metagenomic Analysis. Virulence.

[38] Zimmermann P., Curtis N. (2019). The Effect of Antibiotics on the Composition of the Intestinal Microbiota—A Systematic Review. J. Infect..

[39] Taur Y., Coyte K., Schluter J., Robilotti E., Figueroa C., Gjonbalaj M., Littmann E.R., Ling L., Miller L., Gyaltshen Y. (2018). Reconstitution of the Gut Microbiota of Antibiotic-Treated Patients by Autologous Fecal Microbiota Transplant. Sci. Transl. Med..

[40] Masetti R., D’Amico F., Zama D., Leardini D., Muratore E., Ussowicz M., Fraczkiewicz J., Cesaro S., Caddeo G., Pezzella V. (2022). Febrile Neutropenia Duration Is Associated with the Severity of Gut Microbiota Dysbiosis in Pediatric Allogeneic Hematopoietic Stem Cell Transplantation Recipients. Cancers.

[41] Galloway-Peña J.R., Peterson C.B., Malik F., Sahasrabhojane P.V., Shah D.P., Brumlow C.E., Carlin L.G., Chemaly R.F., Im J.S., Rondon G. (2019). Fecal Microbiome, Metabolites, and Stem Cell Transplant Outcomes: A Single-Center Pilot Study. Open Forum Infect. Dis..

[42] Artacho A., González-Torres C., Gómez-Cebrián N., Moles-Poveda P., Pons J., Jiménez N., Casanova M.J., Montoro J., Balaguer A., Villalba M. (2024). Multimodal Analysis Identifies Microbiome Changes Linked to Stem Cell Transplantation-Associated Diseases. Microbiome.

[43] Biagi E., Zama D., Nastasi C., Consolandi C., Fiori J., Rampelli S., Turroni S., Centanni M., Severgnini M., Peano C. (2015). Gut Microbiota Trajectory in Pediatric Patients Undergoing Hematopoietic SCT. Bone Marrow Transpl..

[44] Azhar Ud Din M., Lin Y., Lyu C., Yi C., Fang A., Mao F. (2025). Advancing Therapeutic Strategies for Graft-versus-Host Disease by Targeting Gut Microbiome Dynamics in Allogeneic Hematopoietic Stem Cell Transplantation: Current Evidence and Future Directions. Mol. Med..

[45] Rashidi A., Ebadi M., Rehman T.U., Elhusseini H., Kazadi D., Halaweish H., Khan M.H., Hoeschen A., Cao Q., Luo X. (2023). Randomized Double-Blind Phase II Trial of Fecal Microbiota Transplantation Versus Placebo in Allogeneic Hematopoietic Cell Transplantation and AML. J. Clin. Oncol..

[46] Golob J.L., Pergam S.A., Srinivasan S., Fiedler T.L., Liu C., Garcia K., Mielcarek M., Ko D., Aker S., Marquis S. (2017). Stool Microbiota at Neutrophil Recovery Is Predictive for Severe Acute Graft vs Host Disease After Hematopoietic Cell Transplantation. Clin. Infect. Dis..

[47] Du Y., He C., An Y., Huang Y., Zhang H., Fu W., Wang M., Shan Z., Xie J., Yang Y. (2024). The Role of Short Chain Fatty Acids in Inflammation and Body Health. Int. J. Mol. Sci..

[48] Xiong R.-G., Zhou D.-D., Wu S.-X., Huang S.-Y., Saimaiti A., Yang Z.-J., Shang A., Zhao C.-N., Gan R.-Y., Li H.-B. (2022). Health Benefits and Side Effects of Short-Chain Fatty Acids. Foods.

[49] Mann E.R., Lam Y.K., Uhlig H.H. (2024). Short-Chain Fatty Acids: Linking Diet, the Microbiome and Immunity. Nat. Rev. Immunol..

[50] Song X., Lao J., Wang L., Liu S. (2024). Research Advances on Short-Chain Fatty Acids in Gastrointestinal Acute Graft- *versus* -Host Disease. Ther. Adv. Hematol..

[51] Rolling T., Zhai B., Gjonbalaj M., Tosini N., Yasuma-Mitobe K., Fontana E., Amoretti L.A., Wright R.J., Ponce D.M., Perales M.A. (2021). Haematopoietic Cell Transplantation Outcomes Are Linked to Intestinal Mycobiota Dynamics and an Expansion of Candida Parapsilosis Complex Species. Nat. Microbiol..

[52] Yang S.-Y., Han S.M., Lee J.-Y., Kim K.S., Lee J.-E., Lee D.-W. (2025). Advancing Gut Microbiome Research: The Shift from Metagenomics to Multi-Omics and Future Perspectives. J. Microbiol. Biotechnol..

[53] de Vos W.M., Tilg H., Van Hul M., Cani P.D. (2022). Gut Microbiome and Health: Mechanistic Insights. Gut.

[54] Masetti R., Zama D., Leardini D., Muratore E., Turroni S., Brigidi P., Pession A. (2021). Microbiome-Derived Metabolites in Allogeneic Hematopoietic Stem Cell Transplantation. Int. J. Mol. Sci..

[55] Yu J., Sun H., Cao W., Han L., Song Y., Wan D., Jiang Z. (2020). Applications of Gut Microbiota in Patients with Hematopoietic Stem-Cell Transplantation. Exp. Hematol. Oncol..

[56] Metafuni E., Di Marino L., Giammarco S., Bellesi S., Limongiello M.A., Sorà F., Frioni F., Maggi R., Chiusolo P., Sica S. (2023). The Role of Fecal Microbiota Transplantation in the Allogeneic Stem Cell Transplant Setting. Microorganisms.

[57] Yoshifuji K., Inamoto K., Kiridoshi Y., Takeshita K., Sasajima S., Shiraishi Y., Yamashita Y., Nisaka Y., Ogura Y., Takeuchi R. (2020). Prebiotics Protect against Acute Graft-versus-Host Disease and Preserve the Gut Microbiota in Stem Cell Transplantation. Blood Adv..

[58] Yazdandoust E., Hajifathali A., Roshandel E., Zarif M.N., Pourfathollah A.A., Parkhideh S., Mehdizadeh M., Amini-Kafiabad S. (2023). Gut Microbiota Intervention by Pre and Probiotics Can Induce Regulatory T Cells and Reduce the Risk of Severe Acute GVHD Following Allogeneic Hematopoietic Stem Cell Transplantation. Transpl. Immunol..

[59] Beckerson J., Szydlo R.M., Hickson M., Mactier C.E., Innes A.J., Gabriel I.H., Palanicawandar R., Kanfer E.J., Macdonald D.H., Milojkovic D. (2019). Impact of Route and Adequacy of Nutritional Intake on Outcomes of Allogeneic Haematopoietic Cell Transplantation for Haematologic Malignancies. Clin. Nutr..

[60] Van Lier Y.F., Davids M., Haverkate N.J.E., De Groot P.F., Donker M.L., Meijer E., Heubel-Moenen F.C.J.I., Nur E., Zeerleder S.S., Nieuwdorp M. (2020). Donor Fecal Microbiota Transplantation Ameliorates Intestinal Graft-versus-Host Disease in Allogeneic Hematopoietic Cell Transplant Recipients. Sci. Transl. Med..

[61] Karimi M., Shirsalimi N., Hashempour Z., Salehi Omran H., Sedighi E., Beigi F., Mortezazadeh M. (2024). Safety and Efficacy of Fecal Microbiota Transplantation (FMT) as a Modern Adjuvant Therapy in Various Diseases and Disorders: A Comprehensive Literature Review. Front. Immunol..

[62] Michailidis L. (2021). Adverse Events of Fecal Microbiota Transplantation: A Metaanalysis of High-Quality Studies. Ann. Gastroenterol..

[63] DeFilipp Z., Bloom P.P., Torres Soto M., Mansour M.K., Sater M.R.A., Huntley M.H., Turbett S., Chung R.T., Chen Y.-B., Hohmann E.L. (2019). Drug-Resistant *E. Coli* Bacteremia Transmitted by Fecal Microbiota Transplant. N. Engl. J. Med..

[64] Wang S., Xu M., Wang W., Cao X., Piao M., Khan S., Yan F., Cao H., Wang B. (2016). Systematic Review: Adverse Events of Fecal Microbiota Transplantation. PLoS ONE.

[65] Biernat M.M., Urbaniak-Kujda D., Dybko J., Kapelko-Słowik K., Prajs I., Wróbel T. (2020). Fecal Microbiota Transplantation in the Treatment of Intestinal Steroid-Resistant Graft-versus-Host Disease: Two Case Reports and a Review of the Literature. J. Int. Med. Res..

[66] Battipaglia G., Malard F., Rubio M.T., Ruggeri A., Mamez A.C., Brissot E., Giannotti F., Dulery R., Joly A.C., Baylatry M.T. (2019). Fecal Microbiota Transplantation before or after Allogeneic Hematopoietic Transplantation in Patients with Hematologic Malignancies Carrying Multidrug-Resistance Bacteria. Haematologica.

[67] Innes A.J., Mullish B.H., Fernando F., Adams G., Marchesi J.R., Apperley J.F., Brannigan E., Davies F., Pavlů J. (2017). Faecal Microbiota Transplant: A Novel Biological Approach to Extensively Drug-Resistant Organism-Related Non-Relapse Mortality. Bone Marrow Transpl..

[68] Ghani R., Mullish B.H., McDonald J.A.K., Ghazy A., Williams H.R.T., Brannigan E.T., Mookerjee S., Satta G., Gilchrist M., Duncan N. (2021). Disease Prevention Not Decolonization: A Model for Fecal Microbiota Transplantation in Patients Colonized with Multidrug-Resistant Organisms. Clin. Infect. Dis..

[69] Neemann K., Eichele D.D., Smith P.W., Bociek R., Akhtari M., Freifeld A. (2012). Fecal Microbiota Transplantation for Fulminant *CLostridium Difficile* Infection in an Allogeneic Stem Cell Transplant Patient. Transpl. Infect. Dis..

[70] De Castro C.G., Ganc A.J., Ganc R.L., Petrolli M.S., Hamerschlack N. (2015). Fecal Microbiota Transplant after Hematopoietic SCT: Report of a Successful Case. Bone Marrow Transpl..

[71] Bluestone H., Kronman M.P., Suskind D.L. (2018). Fecal Microbiota Transplantation for Recurrent Clostridium Difficile Infections in Pediatric Hematopoietic Stem Cell Transplant Recipients. J. Pediatr. Infect. Dis. Soc..

[72] Innes A.J., Mullish B.H., Ghani R., Szydlo R.M., Apperley J.F., Olavarria E., Palanicawandar R., Kanfer E.J., Milojkovic D., McDonald J.A.K. (2021). Fecal Microbiota Transplant Mitigates Adverse Outcomes Seen in Patients Colonized with Multidrug-Resistant Organisms Undergoing Allogeneic Hematopoietic Cell Transplantation. Front. Cell. Infect. Microbiol..

[73] Carlson P.E. (2020). Regulatory Considerations for Fecal Microbiota Transplantation Products. Cell Host Microbe.

[74] Yadegar A., Bar-Yoseph H., Monaghan T.M., Pakpour S., Severino A., Kuijper E.J., Smits W.K., Terveer E.M., Neupane S., Nabavi-Rad A. (2024). Fecal Microbiota Transplantation: Current Challenges and Future Landscapes. Clin. Microbiol. Rev..

[75] Iyama S., Tatsumi H., Shiraishi T., Yoshida M., Tatekoshi A., Endo A., Ishige T., Shiwa Y., Ibata S., Goto A. (2021). Possible Clinical Outcomes Using Early Enteral Nutrition in Individuals with Allogeneic Hematopoietic Stem Cell Transplantation: A Single-Center Retrospective Study. Nutrition.

[76] D’Amico F., Biagi E., Rampelli S., Fiori J., Zama D., Soverini M., Barone M., Leardini D., Muratore E., Prete A. (2019). Enteral Nutrition in Pediatric Patients Undergoing Hematopoietic SCT Promotes the Recovery of Gut Microbiome Homeostasis. Nutrients.

[77] Iyama S., Sato T., Tatsumi H., Hashimoto A., Tatekoshi A., Kamihara Y., Horiguchi H., Ibata S., Ono K., Murase K. (2014). Efficacy of Enteral Supplementation Enriched with Glutamine, Fiber, and Oligosaccharide on Mucosal Injury Following Hematopoietic Stem Cell Transplantation. Case Rep. Oncol..

[78] Riwes M.M., Golob J.L., Magenau J., Shan M., Dick G., Braun T., Schmidt T.M., Pawarode A., Anand S., Ghosh M. (2023). Feasibility of a Dietary Intervention to Modify Gut Microbial Metabolism in Patients with Hematopoietic Stem Cell Transplantation. Nat. Med..

[79] Porcari S., Ng S.C., Zitvogel L., Sokol H., Weersma R.K., Elinav E., Gasbarrini A., Cammarota G., Tilg H., Ianiro G. (2025). The Microbiome for Clinicians. Cell.

[80] Porcari S., Mullish B.H., Asnicar F., Ng S.C., Zhao L., Hansen R., O’Toole P.W., Raes J., Hold G., Putignani L. (2025). International Consensus Statement on Microbiome Testing in Clinical Practice. Lancet Gastroenterol. Hepatol..

[81] Mirzayi C., Renson A., Zohra F., Elsafoury S., Geistlinger L., Kasselman L.J., Eckenrode K., van de Wijgert J., Genomic Standards Consortium, Massive Analysis and Quality Control Society (2021). Reporting Guidelines for Human Microbiome Research: The STORMS Checklist. Nat. Med..

