# Supplementary material for: Unraveling the Intestinal Microbiota Conundrum in Allogeneic Hematopoietic Stem Cell Transplantation: Fingerprints, Clinical Implications and Future Directions
Source: J Clin Med. 2025 Sep 28;14(19):6874. doi: 10.3390/jcm14196874 (PMC12525522; doi:10.3390/jcm14196874)
Supplement: Supplementary file 1 [file jcm-14-06874-s001.zip › jcm-3853037-supplementary.pdf]

Review

Unraveling the Intestinal Microbiota Conundrum in Allogeneic Hematopoietic Stem Cell Transplantation: Fingerprints, Clinical Implications and Future Directions

Alexandre Soares Ferreira Junior,[1] Bianca Fernanda Rodrigues da Silva,[1] Jefferson Luiz da Silva,[1] Mariana Trovao da Silva,[1] Joao Victor Piccolo Feliciano,[2] Iago Colturato,[3] George Maurício Navarro Barros,[4] Phillip Scheinberg,[5] Nelson J Chao,[6] Gislane Lelis Vilela de Oliveira[1*]

1 Department of Genetics, Microbiology and Immunology, Institute of Biosciences, Sao Paulo State University, Botucatu, Sao Paulo, Brazil (alexandre.soares@unesp.br; bianca-fernanda.silva@unesp.br; jefferson.l.silva@unesp.br; mariana.trovao@unesp.br).
2 Fundação Faculdade Regional de Medicina de São José do Rio Preto, São José do Rio Preto, Sao Paulo, Brazil (joao.feliciano@hospitaldebase.com.br).
3 Hospital Amaral Carvalho, Jaú, Sao Paulo, Brazil (iago_colt@hotmail.com).
4 Fundação Pio XII - Hospital de Câncer de Barretos, Barretos, Sao Paulo, Brazil (georgenavarrobr@yahoo.com.br).
5 Division of Hematology, Hospital A Beneficência Portuguesa, São Paulo, Brazil (scheinbp@gmail.com).
6 Department of Medicine, Division of Hematologic Malignancies and Cellular Therapy, Duke University, Durham, North Carolina, United States (nelson.chao@duke.edu).
* Correspondence: gislane.lelis@unesp.br; 55 (14) 3880-0749; Botucatu, Sao Paulo, Brazil.

Supplementary Data

Supplementary Table S1. Key Studies Evaluating the Intestinal Microbiota Prior to allo-HSCT

Author, year<br>N	Sample<br>Timing<br>Control<br>Group	Intestinal Diversity	Microbiota Composition
Peled 2020[1]<br>606	First sample obtained from D-30 to D-6.<br><br>212 participants in the Human Microbiome Project.<br><br>34 healthy adult volunteers.	When compared to the 34 healthy adult volunteers, the 606 patients had lower intestinal diversity ($p < 0.001$).<br><br>When compared to the 212 participants in the Human Microbiome Project, the 606 patients had lower intestinal diversity ($p < 0.001$)<br><br>The diversity between the two cohorts of controls were similar ($p = 0.67$).	The composition of stool samples from the 606 patients were distinct from healthy volunteers as assessed according to the fraction of samples that could be categorized to an enterotype ($p < 0.001$).
Liu 2017[2]<br>57	Samples before conditioning regimen.	When compared to the control group, patients had lower intestinal diversity ($p = 0.0002$)	When compared to the control group, patients had different phylogenetic membership ($p = 0.001$). Patients had increased relative abundancies of facultative

	22 paired HLA-matched sibling donors.		anaerobic bacteria such as *Enterobacteriaceae*, *Lactobacillacea*, *Enterococcaceae* and *Streptococcacea*.
Kusakabe 2020[3]<br>16	Baseline fecal samples collected prior to conditioning regimens (D-8).<br><br>10 healthy volunteers<br>8 patients undergoing auto-HSCT	By using conventional alpha diversity methods, such as Simpson's index or the Shannon-Wiener index, the intestinal diversity appeared to be similar between allo-HSCT and healthy volunteers.	Among the 16 patients undergoing allo-HSCT, 2 received antimicrobial therapy at the time of the baseline fecal specimen collection. In these two patients, major phyla in the gut microbiota were *Actinobacteria* and *Firmicutes.* For the other patients with antimicrobial exposure, the major phyla were *Bacteroidetes*.<br><br>Unweighted UniFrac<br>The unweighted uniFrac distance analysis showed significant differences in microbial components between allo-HSCT and healthy controls ($p < 0.001$).<br><br>Weighted uniFrac<br>The weighted uniFrac distance analysis revealed significant differences in the microbial structure between allo-HSCT and healthy controls ($p < 0.001$)<br><br>In patients undergoing allo-HSCT, the abundance of genera categorized as butyrate-producing bacteria, such as Anaerostipes, Butyricimonas, Coprococcus, Faecalibacterium and Lachnospiraceae, were significantly lower compared to those in the healthy control group.
Sardzikova 2024[4]<br>18$^{\beta}$	Samples were collected prior to allo-HSCT<br><br>14 healthy children	When compared to healthy children, patients undergoing allo-HSCT had significantly lower alpha diversity ($p < 0.001$).	N/A
Mancini 2017[5]<br>96	Samples were collected prior	Alpha diversity was significantly reduced in patients who used the following antibiotics in the 3 months	Use of any antibiotics in the 3 months prior to allo-HSCT was associated with a significant decrease in families with the

	to conditioning regimen (T0).<br><br>N/A	prior to allo-HSCT: 1) any antibiotic (p = 0.001), 2) beta-lactams (p = 0.02), 3) fluoroquinolones (p = 0.05), 4) anti-anerobic therapy* (p = 0.003).	most dominant anaerobic features such as Clostridiaceae (44.15±28.08 vs. 56.44±25.76; p = 0.038), Ruminococcacea (44.13±28.38 vs. 56.47±25.36; p = 0.038) and Veillonellaceae (43.43±28.46 vs. 57.75±24.20; p = 0.016).<br><br>Use of beta-lactams in the 3 months prior to allo-HSCT was associated with a significant decrease in Peptostreptococcaceae (p = 0.011), Ruminococcaceae (p = 0.016), Veillonelaceae (p = 0.040) and Rickinellaceae (p = 0.018). It was also associated with a significant increase in Enterococcaceae (p = 0.045).<br><br>Use of fluoroquinolones in the 3 months prior to allo-HSCT was associated with a significant decrease in Clostridiaceae (p = 0.041)<br><br>Use of anti-anerobic therapy* in the 3 months prior to allo-HSCT was associated with a significant decreased in Clostridiaceae (p = 0.038), Peptostreptococcaceae (p = 0.019), Ruminococcaceae (p = 0.012), Veillonellaceae (p = 0.015) and Rickinellaceae (p = 0.016).
Masetti 2023[6][β]<br>90	Samples were collected prior to allo-HSCT.<br><br>N/A	Higher vs. Lower intestinal diversity prior to allo-HSCT.<br><br>Median antibiotic exposure was significantly longer in patients with lower intestinal diversity (p = 0.028). Additionally, antibiotic therapy prior to allo-HSCT resulted in a significant reduction in alpha diversity (p = 0.043).	Higher vs. Lower intestinal diversity prior to allo-HSCT.<br><br>The higher diversity group was characterized by higher relative abundances of the families Oscillospiraceae, Bacteroidaceae, Rikenellaceae, Ruminococcaceae, Prevotellaceae, Coriobacteriaceae, Christensenellaceae, and Tannerellaceae (p < 0.01). The higher diversity group also had higher proportions of *Bacteroides*, *Dorea*, *Parabacteroides*, *Alistipes*, *Collinsella*, *Coprococcus*, *Roseburia*, *Faecalibacterium*, *Blautia*, and members of the *Christensenellaceae* R-7 group, (*Ruminococcus*) torques group, and

			(*Eubacterium*) coprostanoligenes group ($p < 0.05$).<br><br>The lower diversity group showed an overabundance of Enterococcaceae and Enterobactteriaceae ($p < 0.05$). Additionally, the lower-diversity group was enriched in *Escherichia-Shigella* and *Enterococcus* ($p < 0.05$).
Holler 2014[7]<br>31	Samples were collected prior to allo-HSCT.<br><br>3 donors	N/A	Prior to allo-HSCT vs. Donors<br>At the time of admission, patients showed some loss of dominant commensal strains and an increase in enterococci compared to the donors.
Doki 2017[8]<br>107	Samples were collected 2 weeks prior to the conditioning regimen.<br><br>N/A	Low (<2) vs. Intermediate (2-3) vs. High (>3) Intestinal Diversity<br>Among the 107 patients, 18 (16.8%) patients were in the low diversity group, 48 (44.9%) patients were in the intermediate diversity group, and 41 (38.3%) patients were in the high diversity group. Patients in the low diversity cohort were more likely to had antibiotic exposure before the conditioning regimen.	N/A
Meedt 2022[9]<br>201	Samples were collected at prior to allo-HSCT (D-9 to D-5).<br><br>28 healthy donors	N/A	Prior to allo-HSCT vs. Donors<br><br>There was no difference in copy numbers of fecal Butyryl-CoA/acetate CoA-transferase gene between healthy donors and samples collected prior to allo-HSCT initiation.
Galloway-Pena 2019[10]<br>44	Samples were collected at the time of conditioning	When compared to healthy donors, patients had significantly lower number of observed OTUs ($p < 0.001$) and Shannon index ($p < 0.001$).	Prior to allo-HSCT vs. Donors<br><br>When compared to patients, healthy donors showed a high abundance of *Pseudobutyrivibrio* and *Subdoligranulum*. On the

	18 healthy donors		other side, patients showed higher abundance of *Bacteroides*, *Enterobacter*, *Enterococcus* and *Akkermansia*.
Allo-HSCT = allogeneic hematopoietic stem cell transplantation; N = number of patients included in the analysis; N/A = Not applicable; *Anti-anaerobic therapy = included piperacillin-tazobactam, ticarcillin, meropenem, clindamycin, metronidazole and vancomycin; OTU = Operational taxonomic unit; β = included pediatric patients.			

**Supplementary Table S2. Implications of Intestinal Microbiota Fingerprints Prior to allo-HSCT**		
**Fingerprint**	**Author, year N**	**Findings**
**Enterobacteriaceae abundance >5%**[5]	Mancini 2017[5] 96	↑Sepsis<br>Enterobacteriaceae abundance >5% was associated with increased sepsis risk (HR 6.577; 95% CI 2.246-19.255; $p = 0.021$).
**Lachnospiraceae abundance ≤10%**[5]	Mancini 2017[5] 96	↑All-cause mortality<br>Lachnospiraceae abundance ≤10% was associated with increased all-cause mortality (HR 4.439; 95% CI 2.181-9.035; $p < 0.001$).<br><br>↑Infection-related mortality<br>Lachnospiraceae abundance ≤10% was associated with increased infection-related mortality (HR 7.051; 95% CI 2.007-24.778; $p = 0.006$).<br><br>↑Non-infection-related mortality<br>Lachnospiraceae abundance ≤10% was associated with increased non-infection-related mortality (HR 21.211; 95% CI 4.949-90.915; $p < 0.001$).
**Gram-negative MDR**[5]	Mancini 2017[5] 96	↑Non-infection-related mortality<br>Enteric colonization by Gram-negative MDR was associated with increased non-infection-related mortality (HR 10.280; 95% CI 1.536-68.795; $p = 0.048$).
***Blautia* abundance**[6,11]	Masetti 2023[6]$^{\beta}$ 90	↓aGvHD<br>Higher relative abundance of *Blautia* was protective against subsequent aGvHD development ($p < 0.05$).
	Biagi 2019[11] 36	↓aGvHD<br>When compared to patients who developed GvHD, individuals without aGvHD showed a higher relative abundance of *Blautia* (mainly *Blautia wexlerae*) (8.7% vs. 4.0% [skin GvHD] vs. 1.4% [gut GvHD]; $p = 0.046$). *Blautia* negative association with aGvHD was confirmed by Random Forest analysis ($p = 0.046$)
***Ruminococcus* abundance**[6]	Masetti 2023[6]$^{\beta}$ 90	↓aGvHD<br>Higher relative abundance of *Ruminococcus* was protective against subsequent aGvHD development ($p < 0.05$).
***Streptococcus* abundance**[6]	Masetti 2023[6]$^{\beta}$	↑aGvHD

	90	Higher relative abundance of *Streptococcus* was associated with grade 3 to 4 aGvHD ($p < 0.05$).
***Actinomyces* abundance**[6]	Masetti 2023[6]$^{\beta}$ 90	↑aGvHD<br>Higher relative abundance of *Actinomyces* was associated with grade 3 to 4 aGvHD ($p < 0.05$).
***Lacticaseibacillus* abundance**[6]	Masetti 2023[6]$^{\beta}$ 90	↑aGvHD<br>Higher relative abundance of *Lacticaseibacillus* was associated with grade 3 to 4 aGvHD ($p < 0.05$).
***Rothia* abundance**[6]	Masetti 2023[6]$^{\beta}$ 90	↑aGvHD<br>Higher relative abundance of *Rothia* was associated with grade 3 to 4 aGvHD ($p < 0.05$).
***Firmicutes* abundance**[8]	Doki 2017[8] 107	↑aGvHD<br>Higher abundance of Firmicutes was associated with aGvHD ($p < 0.001$)
***Faecalbacterium* abundance**[8]	Doki 2017[8] 107	↑aGvHD<br>Higher abundance of Faecalbacterium was associated with aGvHD ($p < 0.05$)
***Eubacterium* abundance**[8]	Doki 2017[8] 107	↑aGvHD<br>Higher abundance of *Eubacterium* was associated with aGvHD ($p < 0.05$)
**Fusobacterium abundance**[11]	Biagi 2019[11] 36	↑aGvHD<br>Individuals who developed a more severe aGvHD had a higher relative abundance of Fusobacterium (0% [no GvHD and skin GvHD] vs. 0.05% [gut GvHD]; $p = 0{,}01$). Fusobacterium positive association with aGvHD was confirmed by Random Forest analysis ($p = 0.006$)
Allo-HSCT = Allogeneic hematopoieitic stem cell transplantation; CI = Confidence interval; aGvHD = acute graft versus host disease; HR = Hazard ratio; N = number of patients included in this analysis; MDR = Multidrug resistant; ↓ = decreased; ↑ = increased; β = included only pediatric patients;		

**Supplementary Table S3. Key Studies Evaluating the Dynamics of Intestinal Diversity Over The Allo-HSCT**		
**Author, year N**	**Samples Timing**	**Intestinal Diversity**
Mancini 2017[5] 96	Samples were collected 1) prior to conditioning regimen; 2) D+10, and 3) D+30	Prior to conditioning regimen vs. D+10<br>When compared to samples collected prior to the conditioning regimen, samples at D+10 had a significant decrease in alpha diversity metrics:<br>Shannon index: 4.65±1.36 vs. 3.08±1.77; $p < 0.001$.<br>Inverse Simpson index: 0.85±0.13 vs. 0.65±0.27; $p < 0.001$<br>Observed OTU: 231.61±126.49 vs. 120.23±104.95; $p < 0.0001$<br>Chao1: 477.49±330.06 vs. 229.51±227.31; $p < 0.0001$<br><br>Prior to conditioning regimen vs. D+30

		When compared to samples collected prior to conditioning regimen, samples at D+30 had a significant decrease in alpha diversity metrics:<br>Shannon index: 4.65±1.36 vs. 2.62±1.62; $p < 0.001$.<br>Inverse Simpson index: 0.85±0.13 vs. 0.58±0.26; $p < 0.001$.<br>Observed OTU: 231.61±126.49 vs. 95.77±90.50; $p < 0.001$.<br>Chao1: 477.49±330.06 vs. 180.56; $p < 0.001$.<br><br>D+10 vs. D+30<br>When compared to samples collected at D+10, samples at D+30 had a significant decrease in alpha diversity metrics:<br>Shannon index: 3.08±1.77 vs. 2.62±1.62; $p = 0.020$.<br>Inverse Simpson index: 0.65±0.27 vs. 0.58±0.26; $p = 0.041$.<br>Observed OTU: 120.23±104.95 vs. 95.77±90.50; $p = 0.016$.<br>Chao1: 229.51±227.31 vs. 180.56; $p = 0.027$.
Masetti 2023[6]$^{\beta}$<br>90	Samples were collected 1) prior to allo-HSCT; and 2) at neutrophil engraftment	Prior to allo-HSCT vs. Neutrophil engraftment<br>The intestinal diversity significantly decreased from prior to allo-HSCT to neutrophil engraftment ($p < 0.0001$).
Messina 2024[12]<br>98	Samples were collected once prior to HSCT, weekly until D+30 and then at days D+45, D+90 and D+180	Prior vs. After allo-HSCT<br>In patients undergoing allo-HSCT, there is a decline in alpha diversity of the course of therapy, with the lowest values around D+14. Patients demonstrated a subsequent gradual increase in alpha diversity over time, though it does not normalize to baseline alpha diversity even in samples from D+100.
Taur 2014[13]<br>80	Samples were collected at engraftment	Patients were grouped into 3 levels of microbial diversity based on the inverse Simpson index: high (>4), intermediate (2-4) and low (<2).<br>At the time of stem cell engraftment, high microbial diversity was maintained in 26 (32.5%) patients, intermediate diversity was observed in 20 (25.0%) patient, and low diversity was observed in 34 (42.5%) of patients.<br><br>Patients who developed acute kidney injury, received any of several antibiotics (intravenous vancomycin, metronidazole, B-lactams), or were diagnosed with *C difficile* infection more frequently fell into the low diversity group.
Gu 2022[14]<br>100	Samples were collected at 1) D-10, 2) D-4, and 3) every 4 days until D+60 (or hospital discharge).<br>Samples were grouped into the following timepoints 1)	The intestinal diversity decreased to the lowest at approximately D+12 after allo-HSCT and then increased over time.<br><br>Baseline vs. Pre-HSCT<br>When compared to baseline samples, pre-HSCT samples had a significant decrease in intestinal diversity ($p < 0.05$).

	Baseline (Prior to conditioning); 2) Pre-HSCT (D-4 to D0); 3) Engraftment (D+4 to D+28); and 4) Late Post-HSCT (after D+28)	Baseline vs. Engraftment<br>When compared to baseline samples, samples collected at engraftment had a significant decrease in intestinal diversity ($p < 0.01$).<br><br>Pre-HSCT vs. Engraftment<br>When compared to pre-HSCT samples, samples collected at engraftment had a significant decrease in intestinal diversity ($p < 0.05$).<br><br>Engraftment vs. Late Post-HSCT<br>When compared to samples collected at engraftment, samples from late post-HSCT had a significant increase in intestinal diversity ($p < 0.05$)
Romick-Rosendale 2018[15]<br>42	Samples were collected at prior to allo-HSCT, D0, D+7, and D+14.	Prior to allo-HSCT vs. D+14<br>When compared to pre-HSCT samples, samples collected at D+14 had a significant decrease in intestinal diversity ($p = 0.0028$)
Sardzikova 2024[4]<br>18^{β}	Samples were collected t prior to allo-HSCT, D+7 and D+28.	Prior to allo-HSCT vs. D+7<br>In patients who developed febrile neutropenia, intestinal diversity was significantly decreased in samples collected on D+7 compared to pre-HSCT samples ($p = 0.016$).
Artacho 2024[16]<br>70	Samples were collected at prior to allo-HSCT (4.49 ± 4.24 days before) and engraftment (14.01 ± 1.15 days after)	Prior to allo-HSCT vs. Engraftment<br>Compared to pre–allo-HSCT samples, samples collected at engraftment exhibited a significantly different microbial composition (PERMANOVA $p = 0.002$) and a marked decrease in alpha diversity ($p < 0.0001$). The top significantly different genera between pre allo-HSCT samples and engraftment samples included *Blautia, Clostridium_IV, Roseburia, Oscillibacter, Faecalibacterium, Ruminococcus, Anaerostipes, Intestinimonas, Clostridium_XIVb, Anaerotruncus, Eggerthella, Pseudoflavonifractor, Romboutsia, Intestinibacter, Staphylococcus* and *Lachnospiraceae_UC*. In depth sequencing identified an expansion of three staphylococci species: *S. aureus* ($p < 0.01$), *S. epidermidis* ($p < 0.0001$) and *S. simulans* ($p < 0.0001$).
Allo-HSCT = allogeneic hematopoietic stem cell transplantation; D = day; N = number of patients included in the analysis; OTU = Operational taxonomic unit; β = included pediatric patients.		

Supplementary Table S4. Key Studies Evaluating the Dynamics of SCFA-producing Bacteria and SCFA Levels Over the Allo-HSCT		
**Author, year**<br>**N**	**Samples Timing**<br>**Bacteria**<br>**SCFA**	**Butyrate-producing Bacteria**
Jenq 2015[17]<br>64	D+12<br>*Blautia*<br>N/A	Factors associated with loss of *Blautia* were 1) treatment with antibiotics that inhibit anaerobic bacteria ($p = 0.01$); and 2) total parenteral nutrition for longer durations ($p = 0.01$).
Romick-Rosendale 2018[15]<br>42	Samples were collected at prior to allo-HSCT, D0, D+7, and D+14.<br>N/A<br>Fecal butyrate and propionate	Prior to allo-HSCT vs. D+7<br>Fecal butyrate and propionate levels were significantly decreased at D+7 when compared to baseline (values NR).<br><br>Prior to allo-HSCT vs. D14<br>Fecal butyrate and propionate levels were significantly decreased at D+14 when compared to baseline (values NR).
Meedt 2022[9]<br>201	Samples were collected at prior to allo-HSCT, D0, D+7, D+14, D+21, D+30, and D+90<br>Fecal Butyryl-CoA/acetate CoA-transferase gene copy number	Prior to allo-HSCT vs. D0<br>Levels were significantly decreased at D0 when compared to baseline ($p = 0.01$; r = 0.5).<br><br>Prior to allo-HSCT vs. D+7<br>Levels were significantly decreased at D+7 when compared to baseline ($p = 0.003$; r=0.6).
Haak 2018[18]<br>360	At engraftment<br>SCFA-producing bacteria<br>Fecal butyrate, propionate and acetate	At engraftment, 40% of patients had no detectable butyrate-producing bacteria. A high relative abundance of these bacteria was observed in only 19.2% of patients, while 40.8% had a low relative abundance.
Galloway-Pena 2019[10]<br>44	After HSCT<br>N/A<br>Fecal butyrate	Higher fecal butyrate level was associated with higher Shannon diversity index (r = 0.55; $p < 0.01$) and number of observed OTUs (r = 0.67; $p < 0.01$).

		Patients' samples with low levels of butyrate after HSCT was enriched for *Enterococcus* and *Lactobacillus*.
Artacho 2024[16]<br>70	Samples were collected at prior to allo-HSCT (4.49 ± 4.24 days before) and engraftment (14.01 ± 1.15 days after)<br>N/A<br>Fecal butyrate, propionate and acetate	Compared to pre–allo-HSCT samples, samples collected at engraftment exhibited a significantly decrease in butyrate ($p < 0.0001$), propionate ($p < 0.01$) and acetate ($p < 0.0001$).
Allo-HSCT = allogeneic hematopoietic stem cell transplantation; D = day; N = number of patients included in the analysis; N/A = Not applicable; NR = Not Reported; OTU = Operational taxonomic unit; SCFA = Short chain fatty-acid;		

Supplementary Table S5. Dynamics of Intestinal Domination Over Allo-HSCT

**Intestinal Domination**	**Autor, year<br>N**	**Sample Timing**	**Intestinal Domination Dynamics**
**Any genus**[14]	Gu 2022[14]<br>100	Samples were collected at 1) D-10, 2) D-4, and 3) every 4 days until D+60 (or hospital discharge).<br><br>Samples were grouped into the following timepoints 1) Baseline (Prior to conditioning); 2) Pre-HSCT (D-4 to D0); 3) Engraftment (D+4 to D+28); and 4) Late Post-HSCT (after D+28)	The prevalence of intestinal domination by a certain bacterial genus was about 80% of all patients before the start of conditioning.<br><br>The incidence of intestinal domination increased to approximately 95% of all patients during the engraftment period and then began to decrease.
Enterococcus[12,19–21]	Messina 2024[12]<br>98	Prior to allo-HSCT	2 patients (2.0%) already had *Enterococcus* domination prior to allo-HSCT
		Prior to HSCT (D-30 to D0)<br>Weekly until D+30<br>At days D+45, D+90; D+180 and annually post-transplant.	36% (n=35/98) of patients undergoing allo-HSCT had *Enterococcus* domination in at least one sample.

			The median duration between transplant date and the first stool sample was 22 days (IQR, 6.75–84.25).
	Fujimoto 2024[19]β<br>46	Prior to allo-HSCT<br>Weekly until D+98 or hospital discharge	Among the 317 stool samples, *Enterococcus* domination was presented in 89 (28%).<br><br>Among the 46 patients, Enterococcus domination was observed in 30 (65.2%).<br><br>Stool cultured was performed from samples with *Enterococcus* domination and 30 enterococci were isolated: 11 strains of *Enterococcus faecalis* and 19 strains of *Enterococcus faecium.*
	Stein-Thoeringer 2019[20]<br>1325	Prior to allo-HSCT	Among 1059 samples collected prior to allo-HSCT (between D-30 and D-6), 60 (5.7%) had *Enterococcus faecium* domination.
		From D-20 to D+24	*Enterococcus* domination occurred in up to 65% of patients after allo-HSCT. *Enterococcus faecium* was the dominant species in most patients across all centers.<br><br>*Enterococcus* genus was the most commonly observed to dominate the microbiota in patients from 4 different centers.

		From D0 to D+21	Among 705 patients, *Enterococcus* domination occurred 28.8% (N=203).
		At the time of engraftment (±D+24)	Samples with Enterococcus domination were associated with a loss of *Clostridia spp* (butyrate-producing bacteria).
	Taur 2012[21]<br>94	Prior to allo-HSCT<br>After allo-HSCT (until D+35)	*Enterococcus* was the most frequent dominating genus, occurring in 40.4% (n=38/94) patients.
	Chhabra 2023[22]<br>38	Prior to allo-HSCT<br>After allo-HSCT (until D+21)	*Enterococcus* domination was observed in approximately 30% of patients.
Streptococcus[12,21]	Messina 2024[12]<br>98	Prior to allo-HSCT<br>(D-30 to D0)<br>Weekly until D+30<br>At days D+45, D+90; D+180<br>Annually post-transplant.	*Streptococcus* was the most identified genus responsible for intestinal domination at any timepoint (42% of patients).
	Taur 2012[21]<br>94	Prior to allo-HSCT<br>After allo-HSCT (until D+35)	*Streptococcus* was the second most frequent dominating genus, occurring in 37.2% (n=35/94) patients.
Bacteroides[12]	Messina 2024[12]<br>98	Stools were collected once prior to HSCT (D-30 to D0), weekly until D+30 and then at days D+45, D+90; D+180 and annually post-transplant.	38% of patients undergoing allo-HSCT had *Bacteroides* domination in at least one sample.$^{\Delta}$
Akkermansia[12]	Messina 2024[12]<br>98	Stools were collected once prior to HSCT (D-30 to D0), weekly until D+30 and then at days D+45, D+90; D+180 and annually post-transplant.	28% of patients undergoing allo-HSCT had *Akkermansia* domination in at least one sample.$^{\Delta}$
Blautia[12]	Messina 2024[12]<br>98	Stools were collected once prior to HSCT	28% of patients undergoing allo-HSCT had

		(D-30 to D0), weekly until D+30 and then at days D+45, D+90; D+180 and annually post-transplant.	*Blautia* domination in at least one sample.[Δ]
Lactobacillus[12]	Messina 2024[12]<br>98	Stools were collected once prior to HSCT (D-30 to D0), weekly until D+30 and then at days D+45, D+90; D+180 and annually post-transplant.	28% of patients undergoing allo-HSCT had *Lactobacillus* domination in at least one sample.[Δ]
IQR = Interquartile range; N = Number of patients undergoing allo-HSCT; D = day; Δ = estimated values obtained from a graph; β = intestinal domination defined as relative abundance ≥25%			

References

1. Peled, J.U.; Gomes, A.L.C.; Devlin, S.M.; Littmann, E.R.; Taur, Y.; Sung, A.D.; Weber, D.; Hashimoto, D.; Slingerland, A.E.; Slingerland, J.B.; et al. Microbiota as Predictor of Mortality in Allogeneic Hematopoietic-Cell Transplantation. *N Engl J Med* **2020**, *382*, 822–834, doi:10.1056/NEJMoa1900623.
2. Liu, C.; Frank, D.N.; Horch, M.; Chau, S.; Ir, D.; Horch, E.A.; Tretina, K.; Van Besien, K.; Lozupone, C.A.; Nguyen, V.H. Associations between Acute Gastrointestinal GvHD and the Baseline Gut Microbiota of Allogeneic Hematopoietic Stem Cell Transplant Recipients and Donors. *Bone Marrow Transplant* **2017**, *52*, 1643–1650, doi:10.1038/bmt.2017.200.
3. Kusakabe, S.; Fukushima, K.; Maeda, T.; Motooka, D.; Nakamura, S.; Fujita, J.; Yokota, T.; Shibayama, H.; Oritani, K.; Kanakura, Y. Pre- and Post-serial Metagenomic Analysis of Gut Microbiota as a Prognostic Factor in Patients Undergoing Haematopoietic Stem Cell Transplantation. *Br J Haematol* **2020**, *188*, 438–449, doi:10.1111/bjh.16205.
4. Sardzikova, S.; Andrijkova, K.; Svec, P.; Beke, G.; Klucar, L.; Minarik, G.; Bielik, V.; Kolenova, A.; Soltys, K. Gut Diversity and the Resistome as Biomarkers of Febrile Neutropenia Outcome in Paediatric Oncology Patients Undergoing Hematopoietic Stem Cell Transplantation. *Sci Rep* **2024**, *14*, 5504, doi:10.1038/s41598-024-56242-8.
5. Mancini, N.; Greco, R.; Pasciuta, R.; Barbanti, M.C.; Pini, G.; Morrow, O.B.; Morelli, M.; Vago, L.; Clementi, N.; Giglio, F.; et al. Enteric Microbiome Markers as Early Predictors of Clinical Outcome in Allogeneic Hematopoietic Stem Cell Transplant: Results of a Prospective Study in Adult Patients. *Open Forum Infectious Diseases* **2017**, *4*, ofx215, doi:10.1093/ofid/ofx215.
6. Masetti, R.; Leardini, D.; Muratore, E.; Fabbrini, M.; D'Amico, F.; Zama, D.; Baccelli, F.; Gottardi, F.; Belotti, T.; Ussowicz, M.; et al. Gut Microbiota Diversity before Allogeneic Hematopoietic Stem Cell Transplantation as a Predictor of Mortality in Children. *Blood* **2023**, *142*, 1387–1398, doi:10.1182/blood.2023020026.
7. Holler, E.; Butzhammer, P.; Schmid, K.; Hundsrucker, C.; Koestler, J.; Peter, K.; Zhu, W.; Sporrer, D.; Hehlgans, T.; Kreutz, M.; et al. Metagenomic Analysis of the Stool Microbiome in Patients Receiving Allogeneic Stem Cell Transplantation: Loss of Diversity Is Associated with Use of Systemic Antibiotics and More Pronounced in Gastrointestinal Graft-versus-Host Disease. *Biology of Blood and Marrow Transplantation* **2014**, *20*, 640–645, doi:10.1016/j.bbmt.2014.01.030.
8. Doki, N.; Suyama, M.; Sasajima, S.; Ota, J.; Igarashi, A.; Mimura, I.; Morita, H.; Fujioka, Y.; Sugiyama, D.; Nishikawa, H.; et al. Clinical Impact of Pre-Transplant Gut Microbial Diversity on Outcomes of Allogeneic Hematopoietic Stem Cell Transplantation. *Ann Hematol* **2017**, *96*, 1517–1523, doi:10.1007/s00277-017-3069-8.

9. Meedt, E.; Hiergeist, A.; Gessner, A.; Dettmer, K.; Liebisch, G.; Ghimire, S.; Poeck, H.; Edinger, M.; Wolff, D.; Herr, W.; et al. Prolonged Suppression of Butyrate-Producing Bacteria Is Associated With Acute Gastrointestinal Graft-vs-Host Disease and Transplantation-Related Mortality After Allogeneic Stem Cell Transplantation. *Clinical Infectious Diseases* **2022**, *74*, 614–621, doi:10.1093/cid/ciab500.
10. Galloway-Peña, J.R.; Peterson, C.B.; Malik, F.; Sahasrabhojane, P.V.; Shah, D.P.; Brumlow, C.E.; Carlin, L.G.; Chemaly, R.F.; Im, J.S.; Rondon, G.; et al. Fecal Microbiome, Metabolites, and Stem Cell Transplant Outcomes: A Single-Center Pilot Study. *Open Forum Infectious Diseases* **2019**, *6*, doi:10.1093/ofid/ofz173.
11. Biagi, E.; Zama, D.; Rampelli, S.; Turroni, S.; Brigidi, P.; Consolandi, C.; Severgnini, M.; Picotti, E.; Gasperini, P.; Merli, P.; et al. Early Gut Microbiota Signature of aGvHD in Children given Allogeneic Hematopoietic Cell Transplantation for Hematological Disorders. *BMC Med Genomics* **2019**, *12*, doi:10.1186/s12920-019-0494-7.
12. Messina, J.A.; Tan, C.Y.; Ren, Y.; Hill, L.; Bush, A.; Lew, M.; Andermann, T.; Peled, J.U.; Gomes, A.; Van Den Brink, M.R.M.; et al. *Enterococcus* Intestinal Domination Is Associated With Increased Mortality in the Acute Leukemia Chemotherapy Population. *Clinical Infectious Diseases* **2024**, *78*, 414–422, doi:10.1093/cid/ciab1043.
13. Taur, Y.; Jenq, R.R.; Perales, M.-A.; Littmann, E.R.; Morjaria, S.; Ling, L.; No, D.; Gobourne, A.; Viale, A.; Dahi, P.B.; et al. The Effects of Intestinal Tract Bacterial Diversity on Mortality Following Allogeneic Hematopoietic Stem Cell Transplantation. *Blood* **2014**, *124*, 1174–1182, doi:10.1182/blood-2014-02-554725.
14. Gu, Z.; Xiong, Q.; Wang, L.; Wang, L.; Li, F.; Hou, C.; Dou, L.; Zhu, B.; Liu, D. The Impact of Intestinal Microbiota in Antithymocyte Globulin–Based Myeloablative Allogeneic Hematopoietic Cell Transplantation. *Cancer* **2022**, *128*, 1402–1410, doi:10.1002/cncr.34091.
15. Romick-Rosendale, L.E.; Haslam, D.B.; Lane, A.; Denson, L.; Lake, K.; Wilkey, A.; Watanabe, M.; Bauer, S.; Litts, B.; Luebbering, N.; et al. Antibiotic Exposure and Reduced Short Chain Fatty Acid Production after Hematopoietic Stem Cell Transplant. *Biology of Blood and Marrow Transplantation* **2018**, *24*, 2418–2424, doi:10.1016/j.bbmt.2018.07.030.
16. Artacho, A.; González-Torres, C.; Gómez-Cebrián, N.; Moles-Poveda, P.; Pons, J.; Jiménez, N.; Casanova, M.J.; Montoro, J.; Balaguer, A.; Villalba, M.; et al. Multimodal Analysis Identifies Microbiome Changes Linked to Stem Cell Transplantation-Associated Diseases. *Microbiome* **2024**, *12*, doi:10.1186/s40168-024-01948-0.
17. Jenq, R.R.; Taur, Y.; Devlin, S.M.; Ponce, D.M.; Goldberg, J.D.; Ahr, K.F.; Littmann, E.R.; Ling, L.; Gobourne, A.C.; Miller, L.C.; et al. Intestinal Blautia Is Associated with Reduced Death from Graft-versus-Host Disease. *Biology of Blood and Marrow Transplantation* **2015**, *21*, 1373–1383, doi:10.1016/j.bbmt.2015.04.016.
18. Haak, B.W.; Littmann, E.R.; Chaubard, J.-L.; Pickard, A.J.; Fontana, E.; Adhi, F.; Gyaltshen, Y.; Ling, L.; Morjaria, S.M.; Peled, J.U.; et al. Impact of Gut Colonization with Butyrate Producing Microbiota on Respiratory Viral Infection Following Allo-HCT. *Blood* **2018**, blood-2018-01-828996, doi:10.1182/blood-2018-01-828996.
19. Fujimoto, K.; Hayashi, T.; Yamamoto, M.; Sato, N.; Shimohigoshi, M.; Miyaoka, D.; Yokota, C.; Watanabe, M.; Hisaki, Y.; Kamei, Y.; et al. An Enterococcal Phage-Derived Enzyme Suppresses Graft-versus-Host Disease. *Nature* **2024**, *632*, 174–181, doi:10.1038/s41586-024-07667-8.
20. Stein-Thoeringer, C.K.; Nichols, K.B.; Lazrak, A.; Docampo, M.D.; Slingerland, A.E.; Slingerland, J.B.; Clurman, A.G.; Armijo, G.; Gomes, A.L.C.; Shono, Y.; et al. Lactose Drives *Enterococcus* Expansion to Promote Graft-versus-Host Disease. *Science* **2019**, *366*, 1143–1149, doi:10.1126/science.aax3760.
21. Taur, Y.; Xavier, J.B.; Lipuma, L.; Ubeda, C.; Goldberg, J.; Gobourne, A.; Lee, Y.J.; Dubin, K.A.; Socci, N.D.; Viale, A.; et al. Intestinal Domination and the Risk of Bacteremia in Patients Undergoing Allogeneic Hematopoietic Stem Cell Transplantation. *Clinical Infectious Diseases* **2012**, *55*, 905–914, doi:10.1093/cid/cis580.
22. Chhabra, S.; Szabo, A.; Clurman, A.; McShane, K.; Waters, N.; Eastwood, D.; Samanas, L.; Fei, T.; Armijo, G.; Abedin, S.; et al. Mitigation of Gastrointestinal Graft-*versus*-Host Disease with Tocilizumab Prophylaxis Is

Accompanied by Preservation of Microbial Diversity and Attenuation of Enterococcal Domination. *haematol* **2022**, *108*, 250–256, doi:10.3324/haematol.2022.281309.
